# Pre-oxygenation with high-flow oxygen through the nasopharyngeal airway compared to facemask on carbon dioxide clearance in emergency adults: a prospective randomized non-blinded clinical trial

**DOI:** 10.1007/s00068-023-02418-2

**Published:** 2023-12-26

**Authors:** Jie Li, Bin Liu, Qing-he Zhou, Hua-dong Ni, Ming-juan Liu, Kang Deng

**Affiliations:** 1 Jiaxing University Master Degree Cultivation Base, Zhejiang Chinese Medical University, Jiaxing, 314001 Zhejiang Province China; 2 Department of Anesthesiology and Pain Research Center, The First Hospital of Jiaxing or The Affiliated Hospital of Jiaxing University, Jiaxing, 314001 Zhejiang Province China

**Keywords:** High-flow nasal oxygen, Apneic oxygenation, Carbon dioxide clearance, Airway management, Emergency surgery

## Abstract

**Introduction:**

Before tracheal intubation, it is essential to provide sufficient oxygen reserve for emergency patients with full stomachs. Recent studies have demonstrated that high-flow nasal oxygen (HFNO) effectively pre-oxygenates and prolongs apneic oxygenation during tracheal intubation. Despite its effectiveness, the use of HFNO remains controversial due to concerns regarding carbon dioxide clearance. The air leakage and unknown upper airway obstruction during HFNO therapy cause reduced oxygen flow above the vocal cords, possibly weaken the carbon dioxide clearance.

**Methods:**

Patients requiring emergency surgery who had fasted < 8 h and not drunk < 2 h were randomly assigned to the high-flow group, who received 100% oxygen at 30–60 L/min through nasopharyngeal airway (NPA), or the mask group, who received 100% oxygen at 8 L/min. PaO_2_ and PaCO_2_ were measured immediately before pre-oxygenation (*T*0), anesthesia induction (*T*1), tracheal intubation (*T*2), and mechanical ventilation (*T*3). The gastric antrum’s cross-sectional area (CSA) was measured using ultrasound technology at *T*0, *T*1, and *T*3. Details of complications, including hypoxemia, reflux, nasopharyngeal bleeding, postoperative pulmonary infection, postoperative nausea and vomiting (PONV), and postoperative nasopharyngeal pain, were recorded. The primary outcomes were PaCO_2_ measured at *T*1, *T*2, and *T*3. The secondary outcomes included PaO_2_ at *T*1, *T*2, and *T*3, CSA at *T*1 and *T*3, and complications happened during this trial.

**Results:**

Pre-oxygenation was administered by high-flow oxygen through NPA (*n* = 58) or facemask (*n* = 57) to 115 patients. The mean (SD) PaCO_2_ was 32.3 (6.7) mmHg in the high-flow group and 34.6 (5.2) mmHg in the mask group (*P* = 0.045) at *T*1, 45.0 (5.5) mmHg and 49.4 (4.6) mmHg (*P* < 0.001) at *T*2, and 47.9 (5.1) mmHg and 52.9 (4.6) mmHg (*P* < 0.001) at *T*3, respectively. The median ([IQR] [range]) PaO_2_ in the high-flow and mask groups was 404.5 (329.1–458.1 [159.8–552.9]) mmHg and 358.9 (274.0–413.3 [129.0–539.1]) mmHg (*P* = 0.007) at T1, 343.0 (251.6–428.7 [73.9–522.1]) mmHg and 258.3 (162.5–347.5 [56.0–481.0]) mmHg (*P* < 0.001) at *T*2, and 333.5 (229.9–411.4 [60.5–492.4]) mmHg and 149.8 (87.0–246.6 [51.2–447.5]) mmHg (*P* < 0.001) at *T*3, respectively. The CSA in the high-flow and mask groups was 371.9 (287.4–557.9 [129.0–991.2]) mm^2^ and 386.8 (292.0–537.3 [88.3–1651.7]) mm^2^ at *T*1 (*P* = 0.920) and 452.6 (343.7–618.4 [161.6–988.1]) mm^2^ and 385.6 (306.3–562.0 [105.5–922.9]) mm^2^ at *T*3 (*P* = 0.173), respectively. The number (proportion) of complications in the high-flow and mask groups is shown below: hypoxemia: 1 (1.7%) vs. 9 (15.8%, *P* = 0.019); reflux: 0 (0%) vs. 0 (0%); nasopharyngeal bleeding: 1 (1.7%) vs. 0 (0%, *P* = 1.000); pulmonary infection: 4 (6.9%) vs. 3 (5.3%, *P* = 1.000); PONV: 4 (6.9%) vs. 4 (7.0%, *P* = 1.000), and nasopharyngeal pain: 0 (0%) vs. 0 (0%).

**Conclusions:**

Compared to facemasks, pre-oxygenation with high-flow oxygen through NPA offers improved carbon dioxide clearance and enhanced oxygenation prior to tracheal intubation in patients undergoing emergency surgery, while the risk of gastric inflation had not been ruled out.

**Trial registration:**

This trial was registered prospectively at the Chinese Clinical Research Registry on 26/4/2022 (Registration number: ChiCTR2200059192).

**Supplementary Information:**

The online version contains supplementary material available at 10.1007/s00068-023-02418-2.

## Introduction

Emergency patients fasting for less than the recommended time are at risk of reflux aspiration [1, 2]. Therefore, pre-oxygenation using positive pressure ventilation during induction of anesthesia is assumed to increase this risk [3, 4]. Non-positive end-expiratory pressure (PEEP) pre-oxygenation with a face mask has been widely employed to ensure short periods of well-oxygenated anesthesia induction [5]. Prolonging apneic oxygenation of patients during tracheal intubation after general anesthesia will essentially safeguard the lives of patients [6–8].

High-flow nasal oxygen (HFNO) has been proposed as a technique for apneic oxygenation [6] and has been proven effective for pre-oxygenation to prevent hypoxemia 4–20 min before tracheal intubation [9–12]. In a physiological study of apneic oxygenation during laryngeal surgery, Gustafsson et al. [10] concluded that transnasal humidified rapid insufflation ventilatory exchange, a technique of HFNO, can maintain adequate oxygenation for up to 30 min in patients with mild systemic disease and a BMI < 30. However, its clearing effect on carbon dioxide is debatable. There are some evidences which prove that this technique has an additional impact on carbon dioxide clearance [13, 14], possibly due to the turbulent flow of oxygen from the trachea into the alveoli, clearing the carbon dioxide [11]. However, some clinical trials have shown that HFNO does not have a positive effect on carbon dioxide clearance when compared to a facemask. These trials reported a mean difference of PaCO_2_ ranging from 2.7 to 6.7 mmHg, but no statistical difference was observed [11, 15, 16]. Superimposing high-risk factors of airway obstruction causing patient asphyxia, such as obesity, posterior tongue drop, and soft tissue collapse in the nasopharyngeal cavity, may lower the oxygen flow rate reaching above the voice box than the flow rate at the output end of the cannula after patients losing consciousness, resulting in a change in the gas flow volume. Therefore, we administered high-flow oxygen via the nasopharyngeal airway (NPA) during pre-oxygenation in emergency full-stomach patients to ensure close to the pre-set oxygen flow rate above the vocal cords, and observe carbon dioxide clearance and oxygenation.

In previous studies [9, 17–20], the effect of pre-oxygenation with high-flow oxygen through prong cannula was better than that with facemask. But pre-oxygenation with HFNO was still limited to clinical studies. In this prospective randomized study, to reflect the stability of results, we investigated the utility of pre-oxygenation with high-flow oxygen through NPA comparing with facemask. We hypothesized that there would be significantly lower PaCO_2_ and higher PaO_2_ in emergency surgery patients receiving pre-oxygenation with high-flow oxygen via NPA before tracheal intubation completed compared to a facemask with non-PEEP.

## Methods

The prospective, randomized controlled study was approved on 24/3/2022 by the Ethics Committee of the Affiliated Hospital of Jiaxing University, China (LS2022-KY-069), and registered prospectively to patient enrollment at the Chinese Clinical Research Registry (http://www.chictr.org.cn) on 26/4/2022 (Registration number: ChiCTR2200059192). Written informed consent was obtained from all subjects. It was designed per the principles of the Declaration of Helsinki and conducted according to the Consolidated Standards of Reporting Trials (CONSORT) guidelines at the Affiliated Hospital of Jiaxing University, Jiaxing, China, between April 2022 and March 2023.

### Inclusion and exclusion criteria

The inclusion criteria were patients requiring emergency surgery of all sexes who were 18–60 years old and had fasted < 8 h and not drunk < 2 h with a body mass index (BMI) of 18–35 kg/m^2^, American Society of Anesthesiologists (ASA) Physical Status classes I–III, and New York Heart Association (NYHA) functional classes I–II. The exclusion criteria were pregnancy, risk of severe reflux aspiration (such as intestinal obstruction, diaphragmatic hiatal hernia, and disturbance of consciousness), gastric tube placement, contraindications for NPA placement (nasal polyps, nasopharyngeal masses, hemangiomas, and nasal obstruction, severe maxillary trauma, or skull-base fracture), or inability to provide consent.

### Pre-anesthetic preparation

Before surgery, the patients who met the inclusion criteria were randomized to two groups: receiving pre-oxygenation with high-flow oxygen through NPA (the high-flow group) or a facemask (the mask group). SPSS software version 25 (IBM, Armonk, NY, USA) generated a random sequence by which patients were randomly assigned in a 1:1 ratio to either the high-flow or mask group. This sequence was contained in a sealed envelope, and randomization was concealed until inclusion. Blinding was not feasible beyond this trial stage due to the nature of the intervention.

On entering the operating room, the patients were placed supine on the operating table with their heads elevated at 25° [21], and their vital signs were monitored. An intravenous line was established, and a Ringer’s lactate injection (Taizhou Tianrui Pharmaceutical Co., Ltd, Taizhou, China) was administered before pre-oxygenation. Dexmedetomidine (Dexmedetomidine, Cisen AB, Jining, China) was injected with an intravenous pump of 0.6 μg kg^−1^ for 15 min to relieve anxiety. The anesthetist places an arterial catheter under ultrasound guidance to monitor arterial blood pressure.

### Measurement of CSA

A 2–5 MHz probe of an ultrasonic instrument (GE Medical Systems (China), Wuxi, China) was selected to measure the cross-sectional area of the gastric antrum of patients by permanent anesthesiologists trained in ultrasound. The probe marker points were cephalic and slightly to the right of the median sagittal line below the xiphoid in the upper abdomen. The left lobes of the liver and pancreas were located in front of the gastric antrum. Important marker vessels on the standard sagittal plane of the antrum included the abdominal aorta and superior mesenteric arteriovenous artery. To minimize measurement errors, CSA of the gastric antrum was calculated three times by a single operator by measuring the longitudinal diameter (*D1*) and anteroposterior diameter (*D2*) of the antrum during the intermittent period of gastric antrum contraction using the following formula [22, 23]:

1 $${\text{Antral area}} = \, \left( {\pi \, \times D1 \times D2} \right) \, /{4}.$$

### Pre-oxygenation and anesthesia induction

**Fig. 1:**
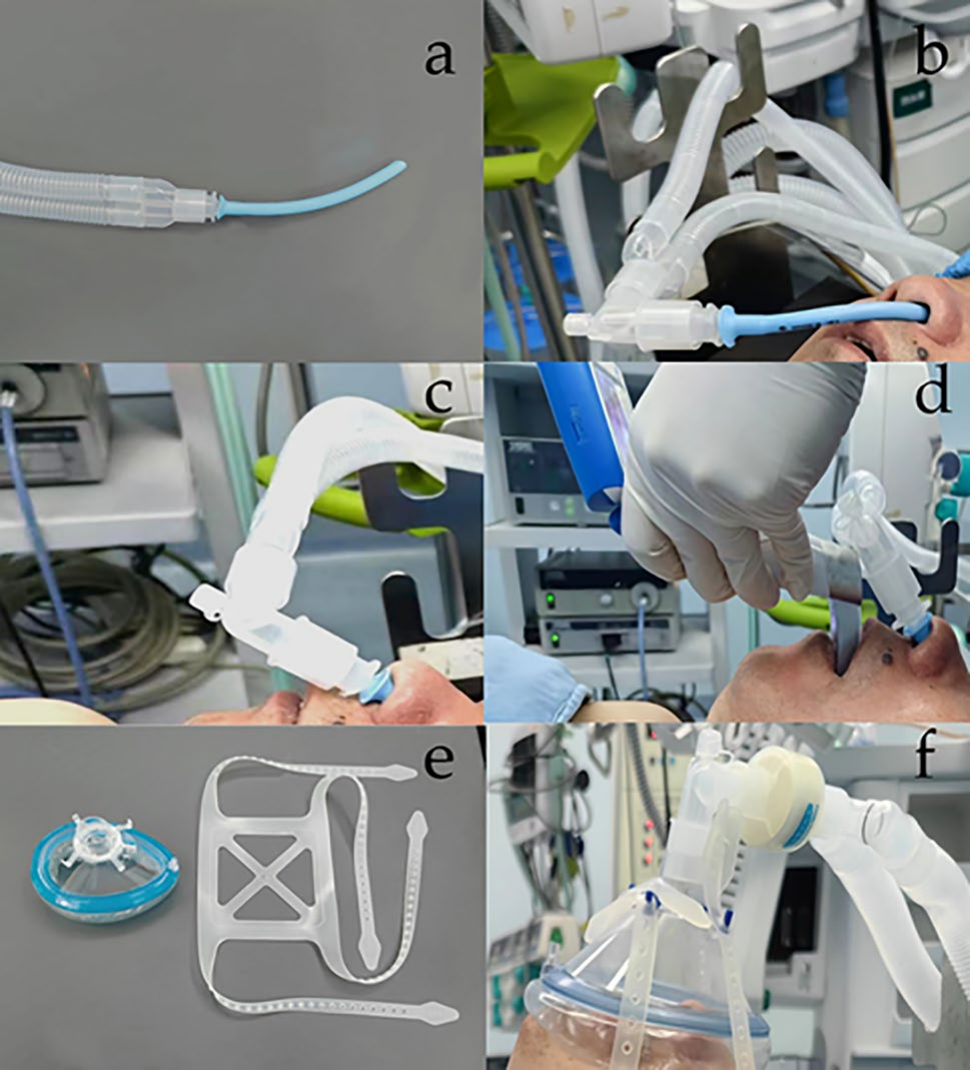
Approaches to pre-oxygenation in two groups

High-flow group: The anesthesiologist administered an appropriate dosage of 2% lidocaine (Lidocaine Hydrochloride®, Kelun AB, Yueyang, China) and ephedrine (Ephedrine®, Shenyang Northeast Yaowei Biological Co. Ltd., Shenyang, China) mixed solution (1:200) into each nostril. The anesthesiologist selected the NPA (Well Lead Medical Co., Ltd, Guangzhou, Chin a) with an internal diameter of 6.0 mm for females and 6.5 mm for males; it was coated with a water-based lubricant and gently inserted into the nostril on one side. Figure 1a shows that NPA was connected beforehand to a respiratory line (Ningbo Huakun Medical Equipment Co., Ltd, Ningbo, China) at the interface of a tracheal tube (Hangzhou Shanyou Medical Equipment Co., Ltd, Hangzhou, China). The insertion depth was limited to the patient’s tolerance (Fig. 1b), temporarily ignoring the target depth (the distance from philtrum to ear tragus) [24]. The high-flow oxygen (PulmoSight™, Mindray, Shanghai, China) was initiated at a flow rate of 30 L/min and an initial oxygen concentration of 100%. The humidity was set to 100% and the temperature to 37 °C (MR850 Respiratory Humidifier, Fisher and Paykel Healthcare, Auckland, New Zealand). Patients were asked to breathe deeply for 3 min. After anesthesia induction and patients’ consciousness loss, NPA was placed at the target depth (Fig. 1c), and the oxygen flow was increased to 60 L/min.

The mask group: the anesthetist selected appropriately sized facemasks with four-head straps (Fig. 1e) that fit tightly over the patient's face (Fig. 1f). Pure oxygen was delivered at 8 L/min through a pressure-free circular circuit (Avance CS2; Datex-Ohmeda, Wisconsin, USA). The patients were instructed to breathe deeply for 3 min. After patients lost consciousness, the anesthesiologist resolved upper airway collapse and closure by jaw thrust [25].

In the high-flow group, NPA was connected to the respiratory line (a); NPA was placed to a depth that patients could tolerate before the anesthesia induction (b); NPA was placed to a target depth after the induction of anesthesia (c); NPA was not removed during tracheal intubation (d). The facemask and four-head belt (Ningbo Huakun Medical Equipment Co., Ltd, Ningbo, China) are shown in e. In the mask group, the facemask was pressed to the patient's face with a four-head belt before tracheal intubation (f). *NPA* nasopharyngeal airway

After 3 min, sufentanil (Sufentanil®, Humanwell AB, Yichang, China) 0.4–0.6 μg kg^−1^, propofol (Propofol®, Fresenius Kabi AB, Graz, Austria) 1.5–2.5 mg kg^−1^, and rocuronium (Esmeron®, N. V. Organon, Oss, The Netherlands) 0.6 mg kg^−1^ were immediately administered intravenously. Both groups were administered oxygen for 2.5 min. Oxygen was still delivered in the high-flow group when tracheal intubation was performed using a visual laryngoscope (Fig. 1d), whereas the facemask was removed in the mask group. Mechanical ventilation was initiated with the ventilator connected, and the end-respiratory carbon dioxide waveform was recorded.

Apnea time was defined as the time from the disappearance of the eyelash reflex to the appearance of the first carbon dioxide waveform after tracheal intubation. Hypoxemia was defined: SpO_2_ ≤ 92% or PaO_2_ < 60 mmHg [26, 27]. Arterial blood would be collected at four time points, immediately before pre-oxygenation (*T*0), anesthesia induction (*T*1), tracheal intubation (*T*2), and mechanical ventilation (*T*3).

### Sample size

The sample size was determined based on a preliminary experiment (11 participants per group) conducted before the formal start of the study. PaCO_2_ after 3 min of pre-oxygenation was used as the outcome index. According to the pre-experimental results, the mean (SD) PaCO_2_ was 33.9 (6.8) mmHg in the high-flow group and 37.9 (7.2) mmHg in the mask group. A total of 100 patients (50 per group) were required to achieve a power of 90% with a type-1 error of 0.05 for detecting the difference between groups, using the PASS software version 11 (NCSS, Kaysville, Utah, USA). Considering a 16% dropout rate, at least 116 patients (58 per group) needed to be included in this study.

### Statistical analysis

Shapiro–Wilk and Kolmogorov–Smirnov tests were used to examine the normality of included variables. Data are presented as mean (SD), numbers (%), or median (IQR [range]) where relevant. The distributions of baseline patient characteristics and outcome variables were compared between groups (high-flow vs mask). Numerical variables were analyzed using an independent samples t-test or the Mann–Whitney *U* test. Categorical variables were compared using Chi-square test, Fisher exact test, or Pearson’s chi-squared test. The primary outcome variables were not all normally distributed, and a non-parametric Mann–Whitney *U* test was used. Statistical significance was set at *P* < 0.05. The data were analyzed using SPSS version 25 (IBM, Armonk, NY, USA).

## Results

**Fig. 2:**
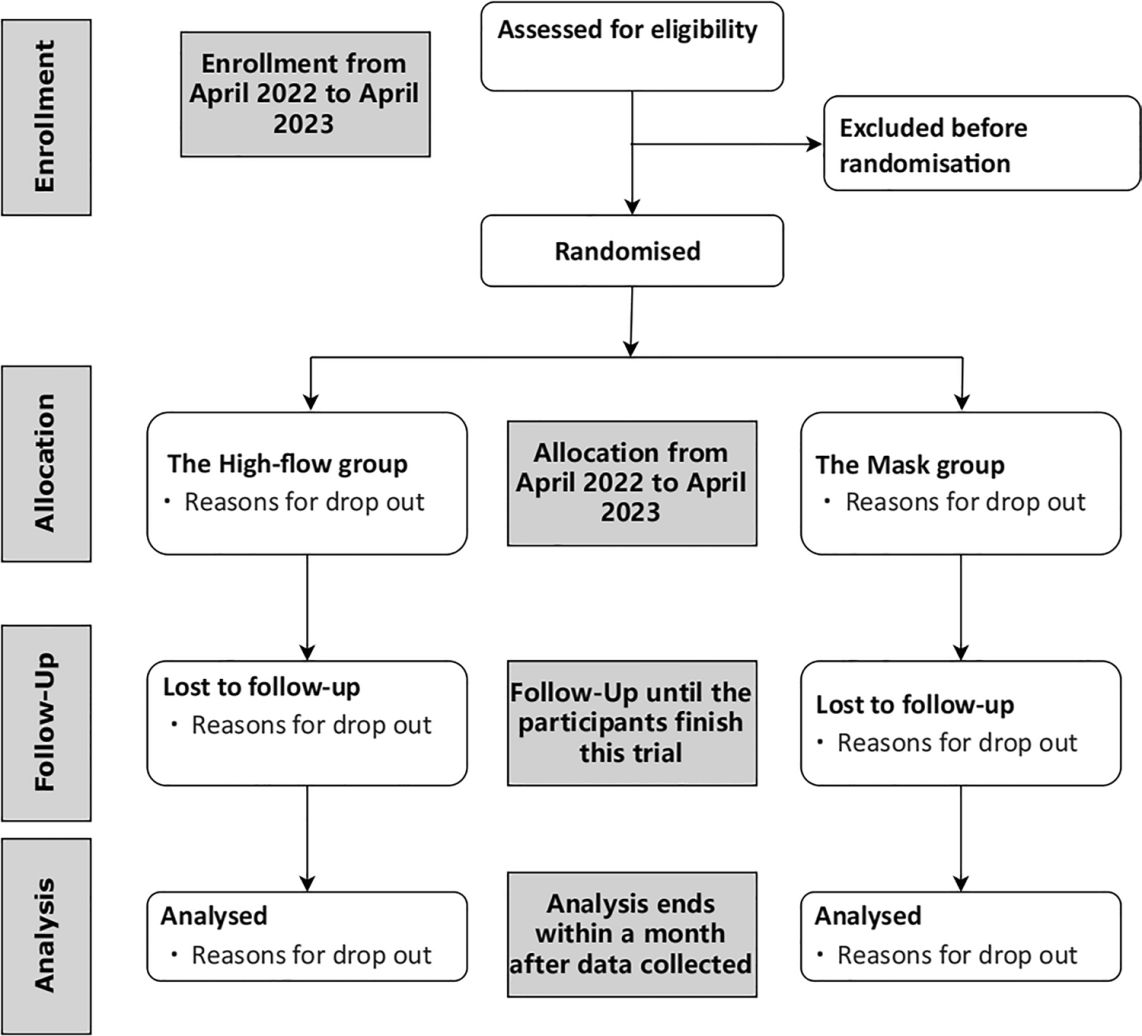
CONSORT flow diagram

The assessed 116 patients were randomly assigned to either the high-flow (*n* = 58) or mask (*n* = 58) groups. All patients underwent randomized pre-oxygenation; one in the mask group dropped out due to a suspected allergic reaction. Finally, 115 patients completed the study protocol (Fig. 2). Patients’ baseline demographic and clinical characteristics in both groups were similar (Table 1).

**Table 1:** Baseline demographic and clinical characteristics of the patients

	High-flow *n* = 58	Mask *n* = 57
Males	34 (58.6%)	32 (56.1%)
Age; years	39.6 (11.0)	39.6 (13.0)
Weight; kg	66.9 (14.3)	64.6 (12.0)
BMI; kg·m^−2^	23.9 (3.8)	23.5 (3.4)
ASA physical status		
I	6 (10.3%)	6 (10.5%)
II	50 (86.2%)	47 (82.5%)
III	2 (3.4%)	4 (7.0%)
NYHA classification		
I	40 (69.0%)	38 (66.7%)
II	18 (31.0%)	19 (33.3%)
Surgical site		
Epigastrium	7 (12.1%)	9 (15.8%)
Hypogastrium	48 (82.8%)	43 (75.4%)
Others	3 (5.2%)	5 (8.8%)
Baseline vital signs		
MAP; mmHg	94.5 (16.0)	97.7 (13.8)
HR; beats.min^−1^	83.6 (13.8)	85.8 (15.1)
SpO_2_; %	99.53(0.8)	99.49(0.8)
Apnea time, s	239.0 (10.6)	239.0 (9.3)
CSA; mm^2^	356.3 (254.4–489.9 [104.6–962.5])	345.0 (287.3–449.7 [74.3–1399.0])

The values are number (proportion), the mean (SD), or median (IQR [range]). *BMI* body mass index, *ASA* American Society of Anesthesiologists, *NYHA* New York Heart Association, *MAP* mean arterial pressure, *HR* heart rate, *CSA* cross-sectional area

### Primary outcomes

The mean (SD) PaCO_2_ was 32.3 (6.7) mmHg in the high-flow group and 34.6 (5.2) mmHg in the mask group (*P* = 0.045) at *T*1, 45.0 (5.5) mmHg and 49.4 (4.6) mmHg (*P* < 0.001) at *T*2, and 47.9 (5.1) mmHg and 52.9 (4.6) mmHg (*P* < 0.001) at *T*3, respectively. PaCO_2_ of patients accepting high-flow oxygen through NPA was statistically lower at *T*1, *T*2, and *T*3 than those pre-oxygenated by facemask (Table 2).

**Table 2:** Clinical characteristics of patients after receiving high-flow oxygen or non-positive pressure facemask pre-oxygenation

	High-flow *n* = 58	Mask *n* = 57	*P*-Value
PaCO_2_, mmHg			
*T*0	35.8 (3.6)	36.3 (3.0)	0.460
*T*1	32.3 (6.7)	34.6 (5.2)	0.045^*^
*T*2	45.0 (5.5)	49.4 (4.6)	< 0.001^*^
*T*3	47.9 (5.1)	52.9 (4.6)	< 0.001^*^
PaO_2_, mmHg			
*T*0	96.3 (13.4)	94.1 (12.9)	0.317
*T*1	404.5 (329.1–458.1 [159.8–552.9])	358.9 (274.0–413.3 [129.0–539.1])	0.007^*^
*T*2	343.0 (251.6–428.7 [73.9–522.1])	258.3 (162.5–347.5 [56.0–481.0])	< 0.001^*^
*T*3	333.5 (229.9–411.4 [60.5–492.4])	149.8 (87.0–246.6 [51.2–447.5])	< 0.001^*^
MAP, mmHg			
*T*1	89.6 (13.9)	91.9 (13.9)	0.372
*T*2	82.9 (13.6)	80.6 (11.0)	0.335
*T*3	77.5 (11.0)	79.9 (13.3)	0.295
HR; beats.min^−1^			
*T*1	81.3 (14.5)	83.5 (16.8)	0.443
*T*2	78.5 (15.5)	77.9 (15.7)	0.849
*T*3	78.0 (16.6)	77.1 (14.5)	0.749
CSA, mm^2^			
*T*1	371.9 (287.4–557.9 [129.0–991.2])	386.8 (292.0–537.3 [88.3–1651.7])	0.920
*T*3	452.6 (343.7–618.4 [161.6–988.1])	385.6 (306.3–562.0 [105.5–922.9])	0.173
Complications			
Hypoxemia	1 (1.7%)	9 (15.8%)	0.019^*^
Reflux	0 (0%)	0 (0%)	-
Nasopharyngeal bleeding	1 (1.7%)	0 (0%)	1.000
Pulmonary infection	4 (6.9%)	3 (5.3%)	1.000
PONV	4(6.9%)	4 (7.0%)	1.000
Nasopharyngeal pain	0 (0%)	0 (0%)	-

Values are number (proportion), the mean (SD), or median (IQR [range]). *T*0 represents immediately before pre-oxygenation. *T*1 represents immediately before anesthesia induction. *T*2 represents immediately before tracheal intubation. *T*3 represents immediately before mechanical ventilation. *PaO*_*2*_ arterial partial oxygen pressure, *PaCO*_*2*_ arterial partial pressure of carbon dioxide, *MAP* mean arterial pressure, *HR* heart rate, *CSA* cross-sectional area, *PONV* postoperative nausea or vomiting

^*^There was difference between groups

### Secondary outcomes

PaO_2_ in the high-flow group was higher at *T*1 (404.5 (329.1–458.1 [159.8–552.9]) mmHg vs. 358.9 (274.0–413.3 [129.0–539.1]) mmHg, *P* = 0.007), *T*2 (343.0 (251.6–428.7 [73.9–522.1]) mmHg vs. 258.3 (162.5–347.5 [56.0–481.0]) mmHg, *P* < 0.001), and *T*3 (333.5 (229.9–411.4 [60.5–492.4]) mmHg vs. 149.8 (87.0–246.6 [51.2–447.5]) mmHg, *P* < 0.001) than that in the mask group. The CSA of the gastric antrum showed no significant differences at *T*1 (371.9 (287.4–557.9 [129.0–991.2]) mm^2^ vs. 386.8 (292.0–537.3 [88.3–1651.7]) mm^2^, *P* = 0.920) and *T*3 (452.6 (343.7–618.4 [161.6–988.1]) mm^2^ vs. 385.6 (306.3–562.0 [105.5–922.9]) mm^2^, *P* = 0.173) between the groups. In both the high-flow and mask groups, there was no significant difference in CSA between *T*1 and *T*3. The CSA measurement was 371.9 (287.4–557.9 [129.0–991.2]) mm^2^ at *T*1 and 452.6 (343.7–618.4 [161.6–988.1]) mm^2^ at *T*3 for the high-flow group (*P* = 0.081), and 386.8 (292.0–537.3 [88.3–1651.7]) mm^2^ at *T*1 and 385.6 (306.3–562.0 [105.5–922.9]) mm^2^ at *T*3 for the mask group (*P* = 0.539), respectively. Mean arterial pressure (MAP) and heart rate (HR) did not significantly differ between the high-flow and mask groups. The occurrence of hypoxemia was lower in the high-flow than in the mask groups (1 (1.7%) vs. 9 (15.8%), *P* = 0.019). The groups did not vary based on other complications, such as reflux, nasopharyngeal bleeding, postoperative pulmonary infection, postoperative nausea or vomiting (PONV), and postoperative nasopharyngeal pain. The secondary outcomes are shown in Table 2.

## Discussion

In emergency patients with a full stomach, tracheal intubation should be performed after induction of anesthesia to achieve optimal tracheal intubation conditions and to avoid reflux aspiration [28]. Compared to rocuronium, suxamethonium may have more effects on cardiovascular events, intragastric pressure, intraocular pressure, fasciculations, etc. [29–31]. However, obtaining sugammadex, an effective antagonist of rocuronium, is challenging due to hospital policies. We believe that excessive doses of rocuronium are inappropriate for this study as they may increase the risk of hypoxemia, hypotension, and delayed anesthesia resuscitation [32]. In our study, a dose of 0.6 mg/kg of rocuronium was considered sufficient for satisfactory tracheal intubation [29, 33]. It is also crucial to wait for the neuromuscular blocking drugs to take effect before performing tracheal intubation. Compared to laryngoscopy initiated 1 min after anesthesia induction by Lodenius et al. [17], we believe that 2 min prior to intubation was acceptable to ensure rocuronium works. Meanwhile, while waiting for tracheal intubation, we can further observe the effect of carbon dioxide clearance and oxygenation in apneic patients in both groups.

**Fig. 3:**
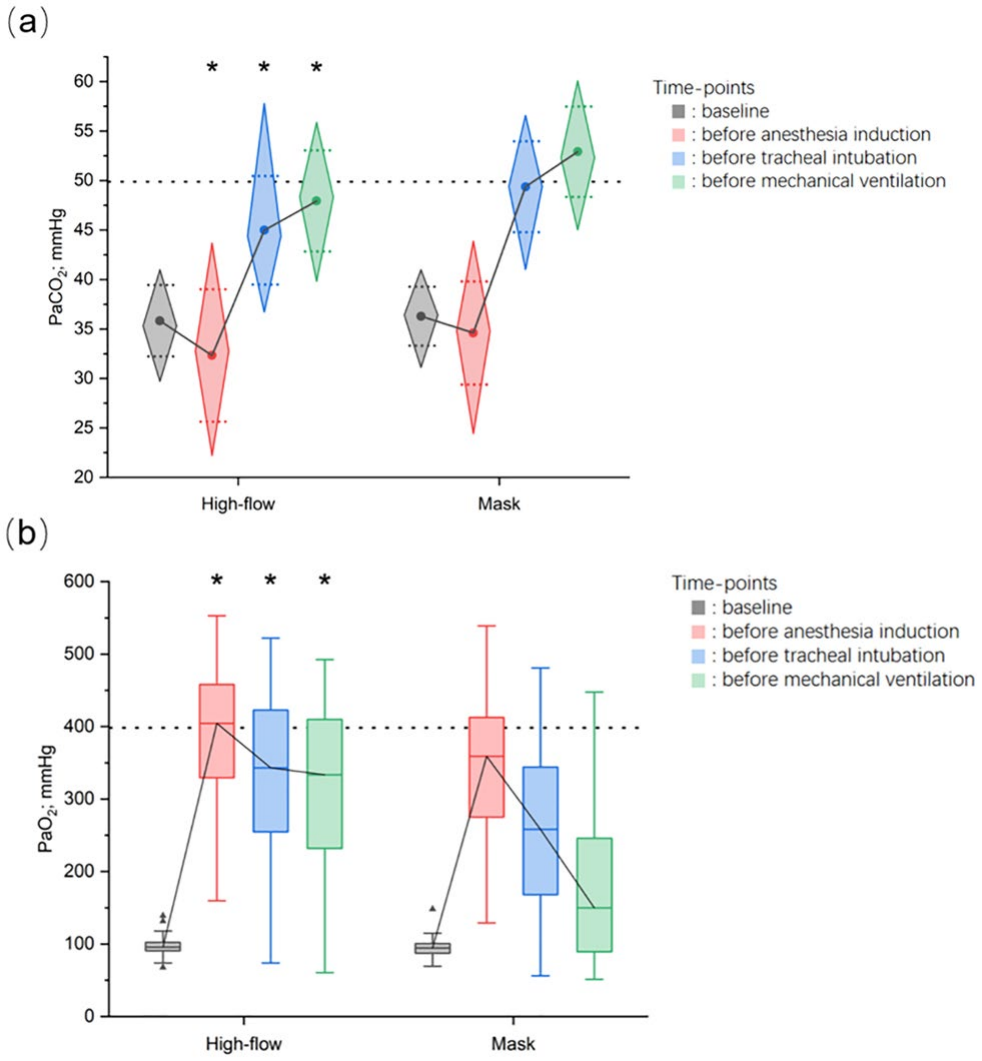
Boxplots of the PaCO_2_ (**a**) and PaO_2_ (**b**) for the high-flow oxygen and mask groups

The significant finding of this study was that PaCO_2_ was lower in the high-flow group than in the mask group during apneic oxygenation (Fig. 3); the magnitude of change in PaCO_2_ during asphyxia (From *T*1 to *T*3) was lesser in patients in the high-flow group (15.6 (5.1) mmHg) than in the mask group (18.3 (4.5) mmHg, *P* = 0.003). HFNO-induced higher minute ventilation and positive airway pressure effects during spontaneous breathing resulted in a more substantial hyperventilation effect in patients of the high-flow group than in those of the mask group. Patients of the high-flow group reported lower PaCO_2_ at *T*1, which had a noticeable impact on PaCO_2_ at *T*2 and *T*3. Therefore, PaCO_2_ at *T*2 and *T*3 do not reflect the CO_2_ clearance effect during respiratory arrest; it is demonstrated by calculating the difference in PaCO_2_ between *T*2 and *T*1, *T*3, and *T*2, and *T*3 and *T*1. The results showed that the PaCO_2_ differences between *T*2 and *T*1, and *T*3 and *T*1 were lower in the high-flow group than the mask group, indicating a delay in the increase of PaCO_2_ during respiratory arrest (Table 3). Hence, HFNO administration using NPA is hypothesized to have a definite CO_2_ clearance effect. Besides the positive airway pressure effect, HFNO has been demonstrated to interact with cardiogenic oscillations, acoustic vortex, and strong turbulence to reduce the rate of CO_2_ accumulation in anesthetized or apneic patients [34]. HFNO can flush residual gas from anatomically invalid cavities, such as the patient's nasal, oral, and pharyngeal cavities, to reduce repeated inhalation of CO_2_ [35]. HFNO facilitates the combination of O_2_ and Hb by the Haldane effect while promoting the release of CO_2_ [36, 37]. However, there is one factor in this study that should not be overlooked. The use of NPA for high-flow oxygen delivery not only eliminates the obstruction factor but also shifts the oxygen output from the nasal vestibule to above the vocal cords, increasing the gas flow and reducing the cross-sectional area of the oxygen delivery line. If gas compression is ignored, the oxygen output through the NPA will have higher air velocity and kinetic energy according to Poiseuille’s law [38]. However, the improved CO_2_ clearance by the increased air supply below the vocal cords is questionable.

**Table 3:** Increase of PaCO_2_ and decline of PaO_2_ after pre-oxygenation in two groups

	High-flow *n* = 58	Mask *n* = 57	*P*-Value
PaCO_2_; mmHg			
From *T*0 to *T*1	3.0 ( – 1.0 to 8.5 [ – 9.3 to 16.7])	0.8 ( – 1.3 to 3.7 [ – 5.3 to 22.5])	0.052
From *T*1 to *T*2	12.6 (5.8)	14.8 (3.9)	0.023^*^
From *T*2 to *T*3	3.2 (1.8–4.6 [ – 10.0 to 15.1])	3.8 (2.5–4.6 [ – 4.0 to 8.2])	0.185
From *T*1 to *T*3	15.6(5.1)	18.3(4.5)	0.003^*^
PaO_2_; mmHg			
From *T*0 to *T*1	300.9 (86.1)	259.5 (80.2)	0.009^*^
From *T*1 to *T*2	37.7 ( – 2.0 to 86.8 [ – 58.2 to 271.0])	83.0 (44.0–163.0 [ – 56.0 to 274.1])	0.002^*^
From *T*2 to *T*3	22.9 ( – 0.3 to 53.4 [ – 43.6 to 114.5])	65.6 (37.2–105.3 [ – 25.3 to 215.4])	< 0.001^*^
From *T*1 to *T*3	72.2 (33.7–142.6 [ – 56.2 to 271.0])	170.3 (116.0–226.3 [ – 33.8 to 359.2])	< 0.001^*^

Values are the mean (SD) or median (IQR [range]). *T*1 represents immediately before anesthesia induction. *T*2 represents immediately before tracheal intubation. *T*3 represents immediately before mechanical ventilation. *PaCO*_*2*_ arterial partial pressure of carbon dioxide, *PaO*_*2*_ arterial partial oxygen pressure

^*^There was difference between groups

In the picture a, the round dots indicate the means, the thick black line connects the means, the diamond-shaped boxes extend to the 95CI and the dotted line of whiskers extend up to SD. In the picture b, the solid horizontal lines indicate the medians, thin black line connects medians, the rectangular boxes extend to the IQR, the solid line of whiskers extends up to 1.5 times the IQR and the outliers beyond are indicated by triangles. *PaCO*_*2*_ arterial partial carbon dioxide pressure, *PaO*_*2*_ arterial partial oxygen pressure, * there was difference at the corresponding time point between groups.

Figure 3 illustrates that patients in the high-flow group had higher PaO_2_ at *T*1, *T*2, and *T*3 than those in the mask group; the mean (SD) increase in PaO_2_ from *T*0 to *T*1 was higher in the high-flow group than in the mask group (300.9 (86.1) mmHg in the high-flow group vs. 259.5 (80.2) mmHg in the mask group, *P* = 0.009). The median (IQR [range]) decrease in PaO_2_ from *T*1 to *T*3 was lesser in the high-flow group than in the mask group (72.2 (33.7–142.6 [ – 56.2 to 271.0]) mmHg in the high-flow group vs. 170.3 (116.0–226.3 [ – 33.8 to 359.2]) mmHg in the mask group, *P* < 0.001). Furthermore, the incidence of hypoxemia was significantly lower in the high-flow group than in the mask group (1.7% vs. 15.8%, *P* = 0.019), possibly due to a combination of factors. Continuous delivery of HFNO generates an appropriate PEEP above the vocal cords that prevents soft tissue collapse in the oropharyngeal and nasopharyngeal cavities and maintains end-expiratory alveolar expansion. This PEEP reduces respiratory work and increases alveolar ventilation during inspiration [34, 35, 39]. The subsequent increase in tidal volume and minute ventilation during spontaneous breathing facilitates the maintenance of numerous open alveoli, potentially allowing a higher oxygen reserve before respiratory arrest [40, 41]. Moreover, apnea oxygenation is predicated on ensuring that the airway between the alveoli, pharyngeal cavity, and oxygen output port is open, permitting continuous oxygen delivery to the nasopharyngeal and oropharyngeal cavities. The airway pressure gradient created by oxygen absorption in the alveoli facilitates oxygen flow from the larger airways to the terminal bronchi and alveoli. The upper airway obstruction is relieved more satisfactorily with HFNO through NPA than with mask pre-oxygenation. This relief increases the gas flow over the vocal fold (almost level with the oxygen flow rate set by the investigator), possibly leading to further enhancement of the PEEP effect [42] and faster airflow at the output end, enhancing the vortex and turbulence effect over the vocal fold [36]. The mask needs to be removed during tracheal intubation in the mask group; in contrast, in the high-flow group, oxygen can still be continuously administered throughout tracheal intubation, presumably reducing the rate of PaO_2_ decline. Therefore, the above mechanisms combinedly ensure better oxygen reserve and oxygenation effects during apnea in patients of the high-flow group. However, further research is required to determine the exact nature of this change.

One of the secondary findings of this study was the absence of significant differences in the CSA of the gastric sinus across different time points between the two patient groups. In recent years, bedside ultrasonography has been used to screen the patient's entire stomach region [43, 44]. The volume of gastric contents, which has been demonstrated to be a vital indicator of the risk of reflux aspiration, was found to positively correlate with sinus CSA measured in the semi-recumbent position in a study of non-pregnant adult patients [44]. While previous studies have generally not found an increased risk associated with HFNO, we had concerns about the potential for gastric inhalation or reflux aspiration when using NPA in this study. As a precaution, we excluded preoperatively as many high-risk patients as possible to minimize the risk of reflux aspiration. Additionally, we used ultrasonic measurements to primarily assess the risk of regurgitant aspiration. Our findings indicate that there were no statistically significant differences in CSA between the two groups at each time point. Furthermore, it is important to note that there were no instances of regurgitant aspiration observed in either group throughout the trial. HFNO ventilation frequently produces low airway pressures. Parke et al. tested high-flow ventilation in healthy volunteers and found that NPA pressure increased by only approximately 1.0 cmH_2_O when the ventilation flow rate was increased by 10 L/min [45]. Another study reported that the mean pressure in the trachea, main bronchus, and pharynx remained below 10.0 cmH_2_O when the ventilation flow rate reached 70 L/min [46]. In contrast, pressure higher than 15 cmH_2_O increases the risk of gastric insufflation [46]. Furthermore, low levels of PEEP generated by HFNO could increase the thoracic pressure, mechanically compress the esophagus, increase the resistance to esophageal outflow, and possibly reduce gastric content reflux [48]. However, it is important to note that HFNO is typically an open system, and the occurrence of gastric insufflation due to airway pressure is not only determined by the pressure value but also influenced by various factors, such as whether the patient has a closed mouth or not, and whether they suffer from gastro-esophageal reflux symptoms. While our study did not find a statistically significant difference in the CSA at T1 and T3 within each group (*P* = 0.081 in the high-flow group and *P* = 0.539 in the facemask group), it is important to consider the potential limitations of our research, such as the lack of power. Therefore, there is a potential risk of increased gastric volume during high-flow oxygen through NPA in clinical practice, and further research is needed to investigate the risk of intragastric distension caused by HFNO during pre-oxygenation. Moreover, it is worth noting that the use of higher flow rates (e.g., 100 to 120 L/min) in clinical settings is uncommon, and it remains unclear whether these higher flow rates lead to a more significant increase in gastric distention.

HFNO is well humidified and is less likely to cause sinus pain, nasopharyngeal mucosal dehiscence, and rhinorrhea [49, 50]. In this study, one patient in the high-flow group and none in the mask group reported nasal bleeding after NPA insertion (1.7% vs. 0%, *P* = 1.000). The occurrence of nasal injury could be minimized by numerous anticipatory measures, including the exclusion of contraindications, selection of soft material and the appropriate size of NPA, adequate lubrication of the nasal mucosa and the outer wall of the NPA, and gentle placement of the NPA. Moreover, there was no significant difference between the two groups in the incidence of postoperative pulmonary infection, PONV, and nasopharyngeal pain.

However, there are a few limitations to this study. Firstly, this is an open trial, as the nature of the intervention did not favor blinding. Secondly, this study could not observe trends in PaO_2_ and PaCO_2_ over an extended period because rapid sequence induction requires minimal respiratory arrest while ensuring adequate sedation and muscle relaxation conditions to reduce the risk of hypoxia. Thirdly, the assessment of significant gas entry into the stomach, facilitated by measuring changes in gastric sinus CSA at different time points, was not continuous and dynamic, permitting possible omissions.

## Conclusions

High-flow oxygen through NPA provides adequate oxygenation and significant carbon dioxide clearance during intubation for anesthesia induction in patients undergoing emergency surgery. However, the risk of gastric inflation had not been ruled out in this study.

### Supplementary Information

Below is the link to the electronic supplementary material.

Supplementary file1 (DOCX 99 KB)

Supplementary file2 (DOCX 806 KB)

Supplementary file3 (DOCX 33 KB)

## Data Availability

The datasets used and/or analyzed during the current study are available from the corresponding author on reasonable request.
