# Supplementary material for: Pre-oxygenation with high-flow oxygen through the nasopharyngeal airway compared to facemask on carbon dioxide clearance in emergency adults: a prospective randomized non-blinded clinical trial
Source: Eur J Trauma Emerg Surg. 2023 Dec 26;50(3):1051–61. doi: 10.1007/s00068-023-02418-2 (PMC11249433; doi:10.1007/s00068-023-02418-2)
Supplement: Supplementary file 1 — Supplementary file1 (DOCX 99 KB) [file 68_2023_2418_MOESM1_ESM.docx]

**Pre-oxygenation with high-flow oxygen through the nasopharyngeal airway compared to facemask on carbon dioxide clearance in emergency adults: a prospective randomized non-blinded clinical trial**

**Research protocol: part 1**

**Project summary**

**The rationale**: Prolonging apneic oxygenation during tracheal intubation is important for emergency patients to reduce risk of hypoxemia. However, for emergency patients not fasting for enough time, who are at risk of reflux-aspiration, inappropriate preoxygenation methods will raise perioperative mortality. High-flow nasal oxygen (HFNO) effectively pre-oxygenates and prolongs apneic oxygenation during tracheal intubation. Despite its effectiveness, the use of HFNO remains controversial due to concerns regarding carbon dioxide clearance. The air leakage and unknown upper-airway obstruction during HFNO therapy cause reduced oxygen flow above the vocal cords, possibly weaken the carbon dioxide clearance.

**Objectives:** The primary objective of this randomized controlled trial was to compare the PaO_2_ in emergency patients with a full stomach when pre-oxygenation was performed using high-flow oxygen through the nasopharyngeal airway (NPA) and facemask. Details of PaO_2_, gastric antrum’s cross-sectional area (CSA) and complications, including hypoxemia, reflux, nasopharyngeal bleeding, postoperative pulmonary infection, postoperative nausea and vomiting (PONV), and postoperative nasopharyngeal pain, were recorded.

**Populations and methods:** Patients requiring emergency surgery who had fasted <8 hours and not drunk <2 hours were randomly assigned to the high-flow group, which received 100% oxygen at 30-60 L/min, or the mask group, which received 100% oxygen at 8 L/min. PaO_2_ and PaCO_2_ were measured immediately before pre-oxygenation (T0), anesthesia induction (T1), tracheal intubation (T2), and mechanical ventilation (T3). CSA was measured using ultrasound technology at T0, T1, and T3.

**Time frame:** This trial began from April 2022 and we expected to finish data collection before April 2023.

**Expected outcomes:** We expected that there would be significantly lower PaCO_2_ values and higher PaO_2_ values in emergency surgery patients receiving preoxygenation via NPA with high-flow oxygen during induction of tracheal intubation compared to a well-sealed facemask.

**General information**

**Protocol title:** Pre-oxygenation with high-flow oxygen through the nasopharyngeal airway compared to facemask on carbon dioxide clearance in emergency adults: a prospective randomized non-blinded clinical trial. The trial was registered at Chinese Clinical Trial Registry on 26/4/2022 (Registration number: ChiCTR2200059192)

**Funder:** This study was supported by A Project Supported by Scientific Research Fund of Zhejiang Provincial Education Department (No. Y202249494), Hangzhou, China and the Construction Project of Anesthesiology Discipline Special Disease Center in Zhejiang (No.201524), Jiaxing, China.

**Investigators:**

Jie Li^1^, M.D., Responsibilities: conceptualization, software, validation, formal analysis, investigation, project administration and writing—original draft preparation.

Bin Liu^1^, M.D., Responsibilities: formal analysis and investigation.

Qing-he Zhou^2^, Ph. D, Professor, Responsibilities: resources, supervision and writing—review and editing.

Hua-dong Ni^2^, Ph. D, Associate Professor, Responsibilities: methodology, validation, data curation and writing—review and editing.

Ming-juan Liu^2^, M.D., Responsibilities: visualization and supervision.

Kang Deng^2*^, M.D., Associate Professor, Responsibilities: conceptualization, methodology, validation, investigation, resources, supervision, project administration, funding acquisition and writing—review and editing.

^1^ Jiaxing University Master Degree Cultivation Base, Zhejiang Chinese Medical University, Jiaxing, Zhejiang Province 314001, China. Telephone number: +86-0573-89997760

^2^ Department of Anesthesiology and Pain Research Center, The First Hospital of Jiaxing or The Affiliated Hospital of Jiaxing University, Jiaxing, Zhejiang Province 314001, China. Telephone number: +86-0573-89997760

*Corresponding author

**Institution involved of this research:** Jiaxing University Master Degree Cultivation Base, Zhejiang Chinese Medical University, Jiaxing, Zhejiang Province 314001, China and Department of Anesthesiology and Pain Research Center, The First Hospital of Jiaxing or The Affiliated Hospital of Jiaxing University, Jiaxing, Zhejiang Province 314001, China

**Rationale & background information**

Emergency patients fasting for less than the recommended time are at risk of re-flux-aspiration [1,2]. Therefore, pre-oxygenation using positive pressure ventila-tion during induction of anesthesia is assumed to increase this risk [3,4]. Non-positive end-expiratory pressure (PEEP) pre-oxygenation with a face mask has been widely employed to ensure short periods of well-oxygenated anesthesia induction [5]. Prolonging apneic oxygenation of patients during tracheal intubation after general anesthesia will essentially safeguard the lives of patients [6-8].

High-flow nasal oxygen (HFNO) has been proposed as a technique for apneic oxygenation [6] and has been proven effective for preoxygenation before tracheal intubation [9-12]. However, its clearing effect on carbon dioxide is debatable. There are some evidences which prove that this technique has an additional im-pact on carbon dioxide clearance [13,14], possibly due to the turbulent flow of oxygen from the trachea into the alveoli, clearing the carbon dioxide [11]. How-ever, some clinical trials shown HFNO has no positive effect on carbon dioxide clearance compared to a facemask [11,15,16]. Superimposing high-risk factors of airway obstruction causing patient asphyxia, such as obesity, posterior tongue drop, and soft tissue collapse in the nasopharyngeal cavity, may lower the oxy-gen flow rate reaching above the voice box than the flow rate at the output end of the cannula after patients losing consciousness, resulting in a change in the gas-flow volume. Therefore, we administered high-flow oxygen via the nasopharyngeal airway (NPA) during preoxygenation in emergency full-stomach patients to ensure close to the pre-set oxygen-flow rate above the vocal cords, and observe carbon dioxide clearance and oxygenation.

In previous studies [9,17-20], the effect of preoxygenation with high-flow oxygen through prong cannula was better than that with facemask. But preoxygenation with HFNO was still limited to clinical studies.

**References**

1. 1. Fawcett WJ, Thomas M. Pre-operative fasting in adults and children: clinical practice and guidelines. Anaesthesia 2019; 74: 83-8.

2. Van de Putte P, Perlas A. Ultrasound assessment of gastric content and volume. Br J Anaesth 2014; 113: 12-22.

3. Lawes EG, Campbell I, Mercer D. Inflation pressure, gastric insufflation and rapid se-quence induction. Br J Anaesth 1987; 59: 315-8.

4. Wagner-Berger HG, Wenzel V, Voelckel WG, et al. A pilot study to evaluate the SMART BAG: a new pressure-responsive, gas-flow limiting bag-valve-mask device. Anesth Analg 2003; 97: 1686-9.5.

5. Cajander P, Edmark L, Ahlstrand R, Magnuson A, de Leon A. Effect of positive end-expiratory pressure on gastric insufflation during induction of anaesthesia when using pressure-controlled ventilation via a face mask: A randomised controlled trial. Eur J Anaesthesiol 2019; 36: 625-32.

6. Lyons C, Callaghan M. Uses and mechanisms of apnoeic oxygenation: a narrative re-view. Anaesthesia 2019; 74: 497-507.

7. Wong CA, Mushambi M. Peri-intubation oxygenation for Caesarean delivery: is there an optimal technique? Br J Anaesth 2022; 129: 468-71.

8. Stolady D, Laviola M, Pillai A, Hardman JG. Effect of variable pre-oxygenation end-points on safe apnoea time using high flow nasal oxygen for women in labour: a mod-elling investigation. Br J Anaesth 2021; 126: 889-95.

9. Mir F, Patel A, Iqbal R, Cecconi M, Nouraei SA. A randomised controlled trial compar-ing transnasal humidified rapid insufflation ventilatory exchange (THRIVE) pre-oxygenation with facemask pre-oxygenation in patients undergoing rapid sequence in-duction of anaesthesia. Anaesthesia 2017; 72: 439-43.

10. Gustafsson IM, Lodenius Å, Tunelli J, Ullman J, Jonsson Fagerlund M. Apnoeic oxy-genation in adults under general anaesthesia using Transnasal Humidified Rapid-Insufflation Ventilatory Exchange (THRIVE) - a physiological study. Br J Anaesth 2017; 118: 610-7.

11. Li Y, Yang J: Comparison of Transnasal Humidified Rapid-Insufflation Ventilatory Exchange and Facemasks in Preoxygenation: A Systematic Review and Meta-Analysis. Biomed Res Int 2022, 2022:9858820.

12. Ayanmanesh F, Abdat R, Jurine A, et al. Transnasal humidified rapid-insufflation ven-tilatory exchange during rapid sequence induction in children. Anaesth Crit Care Pain Med 2021; 40: 100817.

13. Laviola M, Das A, Chikhani M, Bates DG, Hardman JG. Computer simulation clarifies mechanisms of carbon dioxide clearance during apnoea. Br J Anaesth 2019; 122: 395-401.

14. Liew Z, Fenton AC, Harigopal S, Gopalakaje S, Brodlie M, O'Brien CJ. Physiological effects of high-flow nasal cannula therapy in preterm infants. Arch Dis Child Fetal Ne-onatal Ed 2020; 105: 87-93.

15. Booth AWG, Vidhani K, Lee PK, Coman SH, Pelecanos AM, Dimeski G, Sturgess DJ: The Effect of High-Flow Nasal Oxygen on Carbon Dioxide Accumulation in Apneic or Spontaneously Breathing Adults During Airway Surgery: A Randomized-Controlled Trial. Anesth Analg 2021, 133(1):133-41.

16. Hung KC, Ko CC, Chang PC, Wang KF, Teng IC, Lin CH, Huang PW, Sun CK: Effica-cy of high-flow nasal oxygenation against peri- and post-procedural hypoxemia in pa-tients with obesity: a meta-analysis of randomized controlled trials. Scientific reports 2022, 12(1):6448.17.

17. Lodenius A, Piehl J, Ostlund A, Ullman J, Jonsson Fagerlund M: Transnasal humidified rapid-insufflation ventilatory exchange (THRIVE) vs. facemask breathing pre-oxygenation for rapid sequence induction in adults: a prospective randomised non-blinded clinical trial. Anaesthesia 2018, 73(5):564-71.

18. Pillai A, Daga V, Lewis J, Mahmoud M, Mushambi M, Bogod D: High-flow humidified nasal oxygenation vs. standard face mask oxygenation. Anaesthesia 2016, 71(11):1280-3.

19. Rosen J, Frykholm P, Fors D: High-flow nasal cannula versus face mask for preoxy-genation in obese patients: A randomised controlled trial. Acta Anaesthesiol Scand 2021, 65(10):1381-9.

20. Hua Z, Liu Z, Li Y, Zhang H, Yang M, Zuo M: Transnasal humidified rapid insufflation ventilatory exchange vs. facemask oxygenation in elderly patients undergoing general anaesthesia: a randomized controlled trial. Scientific reports 2020, 10(1):5745

**Study goals and objectives**

In this prospective randomized study, we hypothesized that there would be significantly lower PaCO_2_ and higher PaO_2_ in emergency surgery patients receiving preoxygenation with high-flow oxygen via NPA before tracheal intubation completed compared to a facemask with non-PEEP. The primary objectives were PaCO_2_ at three separate time points, i.e. immediately before anesthesia induction (T1), tracheal intubation (T2), and mechanical ventilation (T3). The secondary objectives were PaO_2_ at T1, T2, and T3, CSA of the gastric antrum at T1 and T3, mean arterial pressure (MAP), and heart rate (HR) at each time-point; occurrence of hypoxemia, reflux aspiration, and nasopharyngeal bleeding during tracheal intubation; and postoperative complications such as pulmonary infection, postoperative nausea or vomiting (PONV), and nasopharyngeal pain.

**Study design**

The prospective randomized study was designed per the principles of the Declaration of Helsinki and conducted according to the Consolidated Standards of Reporting Trials (CONSORT) guidelines at the Affiliated Hospital of Jiaxing University, Jiaxing, China. It was expected to be registered prospectively to patient enrolment at the Chinese Clinical Research Registry (<http://www.chictr.org.cn>) before June 2022 and be completed between December 2022 and April 2023.

**Inclusion Criteria:** patients requiring emergency surgery of all sexes who were 18–60 years old and had fasted <8 hours and not drunk <2 hours with a body mass index (BMI) of 18–35 kg/m2, American Society of Anesthesiologists (ASA) Physical Status classes I-III, and New York Heart Association (NYHA) functional classes I-II.

**Exclusion Criteria:** pregnancy, risk of severe reflux-aspiration (such as intestinal obstruction, diaphragmatic hiatal hernia, and disturbance of consciousness), gastric tube placement, contraindications for NPA placement (nasal polyps, nasopharyngeal masses, hemangiomas, and nasal obstruction, severe maxillary trauma, or skull-base fracture), or inability to provide consent.

**Methodology**

**Randomization:** SPSS software version 25 (IBM, Armonk, NY, USA) generated a random sequence by which patients were randomly assigned in a 1:1 ratio to either the high-flow or mask group. This sequence was contained in a sealed envelope, and randomization was concealed until inclusion.

**Blinding:** Blinding was not feasible beyond this trial stage due to the nature of the intervention.

**Stopping rules:** Participants can decline to continue in the study at any stage of the trial. Also, the investigators can terminate the trial if life-threatening events occur during the study, such as severe hypoxemia, hypotension, etc.

**Methods:** On entering the operating room, the patients were placed supine on the operating table with their heads elevated at 25°, and their vital signs were monitored. An intravenous line was established, and a Ringer’s lactate injection (Taizhou Tianrui Pharmaceutical Co., Ltd, Taizhou, China) was administered before pre-oxygenation. Dexmedetomidine (Dexmedetomidine, Cisen AB, Jining, China) was injected with an intravenous pump of 0.6 μg kg^-1^ for 15 min to relieve anxiety. The anesthetist places an arterial catheter under ultrasound guidance to monitor arterial blood pressure.

A 2-5 MHz probe of an ultrasonic instrument (GE Medical Systems (China), Wuxi, China) was selected to measure the cross-sectional area of the gastric antrum of patients by permanent anesthesiologists trained in ultrasound. The probe marker points were cephalic and slightly to the right of the median sagittal line below the xiphoid in the upper abdomen. The left lobes of the liver and pancreas were located in front of the gastric antrum. Important marker vessels on the standard sagittal plane of the antrum included the abdominal aorta and superior mesenteric arteriovenous artery. To minimize measurement errors, CSA of the gastric antrum was calculated three times by a single operator by measuring the longitudinal diameter (D1) and anteroposterior diameter (D2) of the antrum during the intermittent period of gastric antrum contraction using the following formula: Antral area = (π × D1 × D2) /4

The high-flow group: The anesthesiologist administered an appropriate dosage of 2% lidocaine (Lidocaine Hydrochloride®, Kwlun AB, Yueyang, China) and ephedrine (Ephedrine®, Shenyang Northeast Yaowei Biological Co. Ltd., Shenyang, China) mixed solution (1:200) into each nostril. The anesthesiologist selected the NPA (Well Lead Medical Co., Ltd, Guangzhou, Chin a) with an internal diameter of 6.0 mm for females and 6.5 mm for males; it was coated with a water-based lubricant and gently inserted into the nostril on one side. Fig. 1a shows that NPA was connected beforehand to a respiratory line (Ningbo Huakun Medical Equipment Co., Ltd, Ningbo, China) at the interface of a tracheal tube (Hangzhou Shanyou Medical Equipment Co., Ltd, Hangzhou, China). The insertion depth was limited to the patient's tolerance (Fig. 1b), temporarily ignoring the target depth (the distance from philtrum to ear tragus). The high-flow oxygen (PulmoSightTM, Mindray, Shanghai, China) was initiated at a flow rate of 30 L/min and an initial oxygen concentration of 100%. The humidity was set to 100% and the temperature to 37 °C (MR850 Respiratory Humidifier, Fisher and Paykel Healthcare, Auckland, New Zealand). Patients were asked to breathe deeply for 3 min. After anesthesia induction and patients’ consciousness loss, NPA was placed at the target depth (Fig. 1c), and the oxygen flow was increased to 60 L/min.

The mask group: the anesthetist selected appropriately sized facemasks with four-head straps (Fig. 1e) that fit tightly over the patient's face (Fig. 1f). Pure oxygen was delivered at 8 L/min through a pressure-free circular circuit (Avance CS2; Datex-Ohmeda, Wisconsin, USA). The patients were instructed to breathe deeply for 3 min. After patients lost consciousness, the anesthesiologist resolved upper airways collapse and closure by jaw thrust.

After 3 min, sufentanil (Sufentanil®, Humanwell AB, Yichang, China) 0.4-0.6 μg kg^-1^, propofol (Propofol®, Fresenius Kabi AB, Graz, Austria) 1.5-2.5 mg kg^-1^ and rocuronium (Esmeron®, N. V. Organon, Oss, The Netherlands) 0.6 mg kg^-1^ were immediately administered intravenously. Both groups were administered oxygen for 2 min. Oxygen was still delivered in the high-flow group when tracheal intubation was performed using a visual laryngoscope (Fig. 1d), whereas the facemask was removed in the mask group. Mechanical ventilation was initiated with the ventilator connected, and the end-respiratory carbon dioxide waveform was recorded.

Apnea time was defined as the time from the disappearance of the eyelash reflex to the appearance of the first carbon dioxide waveform after tracheal intubation. Hypoxemia was defined: SpO2≤92% or PaO_2_<60 mmHg. Arterial blood would be collected at four time-points, i.e. immediately before pre-oxygenation (T0), anesthesia induction (T1), tracheal intubation (T2), and mechanical ventilation (T3).

**A flow diagram of study design and procedures:**


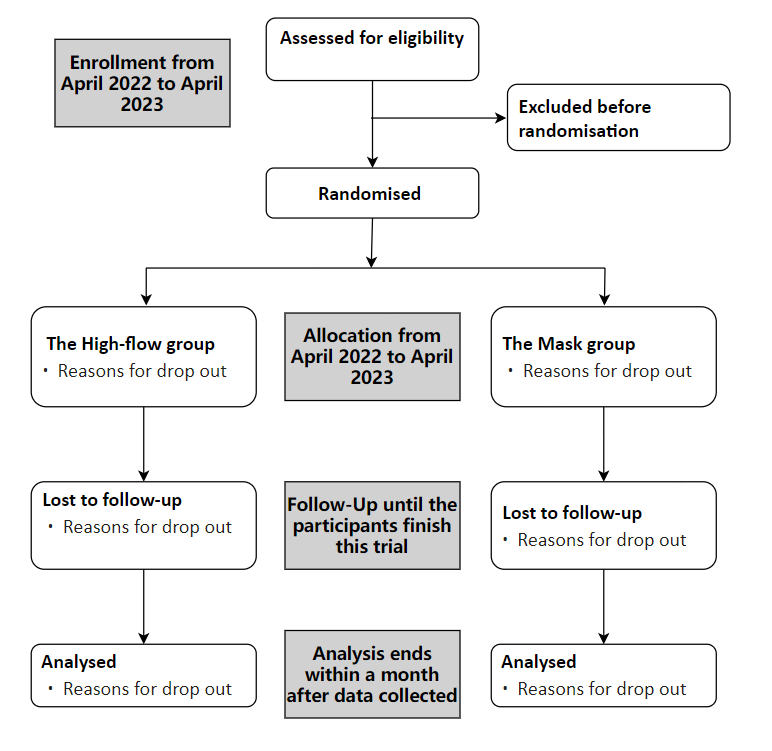


**Safety considerations**

Any event during the trial that endangers the lives of the participants will result in the termination of the study. The participants' identification information will be closely stored in The First Hospital of Jiaxing or The Affiliated Hospital of Jiaxing University, Jiaxing, Zhejiang Province 314001, China. No private information about participants will be released without official legal documents.

**Follow up**

The study would continue until one week after surgery or participant death. During the follow-up period, paticipants' complications will be recorded by visiting or telephone, including hypoxemia, reflux, nasopharyngeal bleeding, postoperative pulmonary infection, postoperative nausea and vomiting (PONV), and postoperative nasopharyngeal pain.

**Data management and statistical analysis**

**Sample size estimation:** The sample size was determined based on a preliminary experiment (11 participants per group) conducted before the formal start of the study. PaCO_2_ after 3 min of pre-oxygenation was used as the outcome index. According to the pre-experimental results, the mean (SD) PaCO_2_ was 33.9 (6.8) mmHg in the high-flow group and 37.9 (7.2) mmHg in the mask group. A total of 100 patients (50 per group) were required to achieve a power of 90% with a type-1 error of 0.05 for detecting the difference between groups, using the PASS software version 11 (NCSS, Kaysville, Utah, USA). Considering a 16% dropout rate, at least 116 patients (58 per group) needed to be included in this study.

**Statistical analysis:** Shapiro–Wilk and Kolmogorov–Smirnov tests were used to examine the normality of included variables. Data are presented as mean (SD), numbers (%) or median (IQR [range]) where relevant. The distributions of baseline patient characteristics and outcome variables were compared between groups (high-flow vs mask). Numerical variables were analyzed using an independent samples t-test or the Mann-Whitney U test. Categorical variables were compared using Chi-square test, Fisher exact test or Pearson’s chi-squared test. The primary outcome variables were not all normally distributed, and a non-parametric Mann-Whitney U test was used. Statistical significance was set at *P*<0.05. The data were analyzed using SPSS version 25 (IBM, Armonk, NY, USA).

**Quality assurance**

During the trial, dedicated researchers performed participant randomization, trial interventions, data collection, and data analysis. They were prohibited from directly communicating about the trial process or results until the first draft of the manuscript was written. There were also monitors throughout the trial who randomly checked the trial progress and data collection.

**Expected outcomes of the study**

In this prospective randomized controlled study, we expected that there would be significantly lower PaCO_2_ values and higher PaO_2_ values in emergency surgery patients receiving preoxygenation via NPA with high-flow oxygen during induction of tracheal intubation compared to a well-sealed facemask.

Emergency patients tend to have poor oxygen reserves, which puts them at risk of experiencing hypoxemia before tracheal intubation is completed. Inappropriate preoxygenation will greatly increase the risk of reflux aspiration in emergency patients due to extra factors such as insufficient fasting and drinking time, uncertainty of gastric contents and so on. With this study, we sought to provide a novel and effective way of preoxygenation for patients undergoing emergency surgery that would provide adequate oxygen reserves without increasing the risk of regurgitant aspiration. At the same time, the debate on whether HFNO has additional carbon dioxide clearance is supported by the research data, which will provide a reference value for future studies related to HFNO.

**Dissemination of results and publication policy**

If necessary, the results of the study will be disseminated in a legal form in the medical-related field according to local laws. Jie Li is the principal investigator of the study and primarily responsible for paper's publication. All co-authors and people who will have assisted in the study will be acknowledged in the paper.

**Duration of the project**

March 2022: Completion of preliminary trial protocol and related pre-experiments

April 2022: Study protocol approved by ethics committee and registration completed at the Chinese Clinical Research Registry.

From May 2022 to March 2023: Conducting experiments, collecting data, analyzing data, writing manuscripts and preparing the submission.

**Problems anticipated**

The occurrence of emergency surgery is randomized and not all patients are suitable or willing to participate in this study. Coupled with the fact that the study period coincided with the spread of Corona Virus Disease 2019 (COVID-19), our participant recruitment efforts were quite difficult. The condition of emergency patients is rapidly changing and various crisis events, such as shock, may occur in the perioperative period, which would stop the trial midway. This is the first time we have used high-flow oxygen therapy via NPA in emergency patients with a full stomach, and it is unclear whether this leads to the development of gastric insufflation. However, we used ultrasound techniques to measure the cross-sectional area of the gastric sinus to indirectly determine whether gastric insufflation occurred. If significant gastric insufflation occurs, we will terminate the trial.

**Project management**

For Conceptualization, K.D. and J.L.; methodology, K.D. and J.L.; software, J.L.; validation, J.L., H.-D.N. and K.D.; formal analysis, J.L. and B.L.; investigation, J.L., B.L. and K.D.; resources, Q.-H.Z. and K.D.; data curation, H.-D.N.; visualiza-tion, M.-J.L.; supervision, Q.-H.Z., M.-J.L. and K.D.; project administration, J.L. and K.D.; funding acquisition, K.D.; writing—original draft preparation, J.L.; writing—review and editing, K.D., H.-D.N. and Q.-H.Z. All authors must have read and agreed to the published version of the manuscript before submission.

**Ethics**

All procedures performed in studies involving human participants were in ac-cordance with the ethical standards of the Affiliated Hospital of Jiaxing Universi-ty and with the 1964 Helsinki Declaration and its later amendments or compara-ble ethical standards. The study was approved by the Ethics Committee of the Affiliated Hospital of Jiaxing University, Jiaxing, China (LS2022-KY-069).

At the time of participant recruitment, the details of the trial and the possible risks were communicated in detail by the investigator and written consent was obtained from the patient before inclusion in the study. Patients must be informed beforehand that they will not receive any financial benefit for participating in the study.

**Informed consent forms**

**English Version**

**Informed Consent Form for Scientific Research Projects**

Dear patients:

We invite you to participate in a study approved by the Ethics Committee of the Affiliated Hospital of Jiaxing University, China, on "Comparison of the efficacy and safety of a modified trans-nasal humidified rapid inflation exchange technique (THRIVE) and a conventional mask during rapid sequential anesthesia induction in emergency patients. The study will be conducted at the Affiliated Hospital of Jiaxing University and an estimated 150 subjects will volunteer to participate. This study has been reviewed and approved by the Ethics Committee of the Affiliated Hospital of Jiaxing University.

Some of the content covered herein is subject to regulatory requirements and has been reviewed and approved by the ethics committee to protect the rights of the patients participating in the study. Why is this study being conducted?

**Background of the study:**

Despite continuous developments and innovations in anesthesiology and dramatic improvements in patient safety in the perioperative period, many adverse events still occur during anesthesia, of which hypoxemia accounts for approximately 20% of these adverse events. Due to the use of muscle relaxants and other anesthetic drugs, patients undergoing general anesthesia inevitably experience a period of asphyxia before tracheal intubation. To reduce the risk of decreased arterial oxygen saturation during intubation, the anesthesiologist must take effective preoxygenation measures to increase the patient's oxygen stores to supplement the oxygen level during intubation.

To reduce the risk of decreased arterial oxygen saturation during intubation, the anesthesiologist must take effective preoxygenation method to increase the patient's oxygen stores to supplement the oxygen supply during intubation. In clinical practice, physicians are often confronted with emergency full stomach patients who have not fasted for enough time, and the uncertainty of gastric contents may lead to reflux of gastric contents. The uncertainty of gastric contents may lead to reflux aspiration of gastric contents, and once additional gas enters the stomach, the risk of reflux aspiration is greatly increased, threatening the patient's perioperative safety. In the traditional mask preoxygenation method, the patient is completely dependent on the inhalation of high concentration oxygen to store oxygen while awake, and there is basically no effective gas inhalation between the administration of anesthetic drugs and the completion of tracheal intubation. The newest oxygen delivery technology, THRIVE, has been shown to provide continuous, uninterrupted oxygen supply before induction of anesthesia, after induction of anesthesia, and during tracheal intubation, while also increasing carbon dioxide elimination, further safeguarding. This will further ensure the safety of emergency patients. However, the efficacy and safety of this new technology compared to conventional mask preoxygenation needs to be confirmed through further studies.

**Objectives of the study:**

(1) To demonstrate that the modified THRIVE as a preoxygenation technique can be effectively used for induction of anesthesia and intubation in emergency full stomach patients, and can compensate for the deficiencies of the traditional face mask

(2) In this study, we propose to use ultrasound visualization to measure the cross-sectional area of the gastric sinus at different points in order to assess gastric air intake, and then to investigate whether the modified THRIVE increases the risk of gastric distention and regurgitant aspiration compared with conventional mask preoxygenation in emergency full stomach patients.

**Scope of the trial:**

All patients participating in this study are from our hospital who require general anesthesia for tracheal intubation in the emergency department. How was the study conducted?

Patients in the study were divided into a test group using a random number table and a control group using a modified THRIVE for preoxygenation during induction of general anesthesia. The control group was preoxygenated during general anesthesia induction intubation using a normal mask. We will record the results of blood gas analysis after the patient is admitted to the room, after preoxygenation, before tracheal intubation and after tracheal intubation. We will also measure the cross-sectional area of the patient's gastric sinuses using ultrasound techniques after admission, before and after intubation to assess the patient's gastric air intake. Vital signs such as blood pressure, heart rate, and pulse oximetry are monitored at all times during the operation. In addition, the investigators are concerned about hypoxemia, nausea and vomiting, regurgitant aspiration, nasopharyngeal discomfort, and air pressure, nasopharyngeal discomfort, pneumatic injuries, and other complications.

**What should I do in the study?**

This study is reviewed and approved by the Ethics Committee of Jiaxing First Hospital, you will be required to do some project-related tests at our hospital and to report any changes or discomfort at any time during the study. You will need to report any changes or discomfort during the study.

**How will participating in the study affect my life?**

You may feel that these tests are inconvenient and require special arrangements. In addition, some tests such as arterial puncture placement may also make you feel uncomfortable. Some tests such as nasopharyngeal ventilation tubes may cause nasal bleeding, but this is a common and important monitoring tool during emergency surgery and will keep you safe during the procedure. However, we will lubricate the nasal cavity and catheter in advance to significantly reduce the incidence of this, and stop the bleeding immediately with general pressure management if it occurs. If you have any questions about the tests and procedures in the study. If you have any questions about the tests and procedures in the study, you may consult with the study physician.

You are not allowed to use analgesics, sedatives, antihypertensives, etc. during the study without informing the study staff. Your study doctor will inform you that during the study period. You need to cooperate with your doctor in the treatment and handling of the study, and inform your doctor of your condition and reactions in a timely and truthful manner, and we will give you immediate feedback and assistance.

**What are the risks and adverse effects of my participation in this study?**

You may have an adverse reaction during the study. We will monitor all patients in the study for any adverse reactions. If you experience any adverse reactions. Please consult with your study doctor promptly.

Currently, the project may cause discomfort: 1, hypoxemia; 2, rhinorrhea; 3, regurgitant aspiration, etc.

You should tell your family or close friends that you are participating in a study entitled "Comparison of the efficacy and safety of a modified THRIVE and a conventional mask during rapid sequential anesthesia induction in emergency patients. They can be aware of the events described above. If they have questions about your participation in the study, you can tell them. If they have questions about your participation in the study, you can tell them how to contact your study physician.

**What benefits will I get from this study?**

Participation in this study will enable you to reduce the incidence of hypoxemia during induction of anesthesia and intubation, reduce the level of carbon dioxide accumulation, and reduce the impact of apnea on your physiological and reduce the disturbance of your physiological status. In case of difficult intubation, it will also extend the safe duration of intubation and better protect your life.

**What will I be paid for participating in this study?**

You will not receive any payment for participating in this study, nor will you receive a reduction in the cost of surgical anesthesia for participating in this study. However, any additional tests and supplies (abdominal ultrasound, high-flow anesthesia, etc.) that are involved in this study. However, any additional tests and supplies involved in this study (abdominal ultrasound, high-flow oxygen ventilator and its breathing loop, nasopharyngeal airway, lidocaine hydrochloride gel, paraffinoil), will be provided free of charge.

What happens if I suffer damage during my participation in the study? How can damage be avoided and what is the treatment if damage occurs?

Theoretically, there is still a risk of hypoxemia and related adverse effects in patients with nasopharyngeal airway and HTHRIVE oxygenation. However, our anesthesiologists. However, our anesthesiologists are experienced in intraoperative management and can handle hypoxemia individually according to the specific situation. The possibility of long-term hypoxemia is very small. However, in the case of patients with intraoperative combined hypotension and severe water-electrolyte acid-base imbalance may make anesthesia management. Therefore, we will strengthen circulatory monitoring during the study period and keep vasoactive drugs on hand to prevent intraoperative complications. At the same time, our study team will be available to answer your questions. We will provide you with prompt and thoughtful medical services. We will also develop detailed protocols and follow strict protocols. If your health does experience study-related damage as a result of participating in this study, please notify the study physician immediately and they will be responsible for taking appropriate treatment measures for you. The Affiliated Hospital of Jiaxing University will bear the cost of treatment and provide you with appropriate financial compensation in accordance with the relevant national regulations. For medical malpractice or for failure to follow the study protocol procedures. The sponsor will not compensate you for injuries caused by your own poor diet or lifestyle practices. Even if you have signed this informed consent form, you retain all of your legal rights.

**Is my personal information confidential?**

Your medical records will be kept at the hospital and the investigator, study authorities, and ethics committee will be allowed access to your medical records. Any public reports regarding the results of this study will not disclose your personal identity in any public report of the results of the study. We will protect the privacy of your personal medical information to the extent permitted by law. By signing this informed consent form, you are consenting to the use of your personal and medical information in the ways described above.

Do I have to participate in the study? What are the alternatives?

Participation in this study is completely voluntary and you may refuse to participate in the study or choose to withdraw from the study at any time during the study without any reason. This decision will not affect your normal treatment.

If you decide to withdraw from the study, please notify your study doctor in advance. For your safety, you may be asked to undergo tests that are necessary to protect your health.

We have also developed alternative options for you, such as performing conventional pressurized mask preoxygenation and high-frequency jet ventilation for induction of anesthesia and oxygenation during intubation. Of course, there are risks associated with these alternatives, such as hypoxemia, hypercapnia, regurgitant aspiration, etc.

**Subjects' Consent Statement:**

I have read the above description of this study and am fully aware of the possible risks and benefits of participating in this study. I am voluntarily consenting to participate in the clinical study described herein.

I agree□ do not agree□ to the use of my medical records and pathology specimens in studies other than this study.

Informed Consent Form Version: 1.0 March 10, 2022

Subject's Signature: Date:

Name in block letters:

Subject's contact phone number: Mobile phone number:

Legal representative's signature (if any): Date:

Legal representative's name in block letters:

Investigator's Statement: I confirm that I have explained to the patient the details of this study, in particular the possible risks and benefits of participating in this study.

Investigator's Signature: Date:

Investigator's name in block letters: Jie Li Kang Deng

Investigator's contact number: 13013984418; 13736446844

Clinical Research and New Medical Technology Ethics Committee Sub-Committee Contact: Jiaxing First Hospital, 1882 South Zhonghuan Road, Jiaxing, Zhejiang, China

**Chinese Version**

**科研项目知情同意书**

尊敬的患者：

我们邀请您参加嘉兴市第一医院（嘉兴学院附属医院）伦理委员会批准开展“改良型经鼻湿化快速充气交换技术（THRIVE）与传统面罩在急诊患者快速顺序麻醉诱导期间的疗效及安全性比较”的研究。本研究将在嘉兴市第一医院开展，估计将有150名受试者自愿参加。本研究已经得到嘉兴市第一医院伦理委员会的审查和批准。

本文涵盖的部分内容由法规要求而定，并且为了保护参加研究的患者的权益，本文经伦理委员会审核并同意。 为什么要开展本项研究？

**研究背景：**

尽管麻醉学不断发展创新，患者围术期的安全性已有了巨大改善，但麻醉过程中仍有许多不良事件发生，其中低氧血症大约占这些不良事件的20%。由于肌肉松弛剂等麻醉药物的使用，全麻患者气管插管前不可避免要经历一段窒息时间，为了降低插管期间动脉氧饱和度降低的风险，麻醉医师必须采取有效的预充氧措施，提高患者氧气储备以补充插管过程中的氧供。在临床实践中，医生经常面对禁食时间不足的急诊饱胃病人，胃内容物的不确定性可能导致胃内容物的反流误吸的发生，一旦再有额外的气体进入胃部，将大大增加反流误吸的风险，威胁患者的围术期安全。传统面罩预给氧方式，完全依靠患者清醒时吸入高浓度氧气储氧，在给予麻醉药物后至完成气管插管这段时间是基本没有有效气体吸入的。目前已有研究证明最新给氧技术——经鼻湿化快速充气交换技术（THRIVE）可在麻醉诱导前、麻醉诱导后、气管插管过程中可以继续、不中断供氧，同时亦能增加二氧化碳排除，将进一步保障急诊患者的安全。但目前该项新技术相比于传统面罩预充氧，其疗效及安全性尚需要通过进一步研究证实。

**研究的目的：**

（1） 拟证明改良型THRIVE作为预充氧技术能有效应用于急诊饱胃患者的麻醉诱导及插管，并能够弥补传统面罩预给氧方式的不足，进一步增加急诊饱胃患者快速顺序诱导插管期间的安全保障。

（2） 本研究拟利用超声可视化技术测量不同时点胃窦部横截面积等指标，以评估胃进气情况，继而探讨在急诊饱胃患者手术中，改良型THRIVE相比于传统面罩预给氧是否会增加胃胀气及反流误吸的风险。

**试验范围：**所有参与本研究患者均来自我院急诊需行气管插管全麻手术的患者。 该研究是怎样进行的？

研究中采用随机数字表法将患者分为试验组与对照组，试验组采用改良型THRIVE行全麻诱导插管期间预充氧；对照组采用普通面罩行全麻诱导插管期间预充氧。我们将在患者入室后、预充氧后、气管插管前、气管插管后记录下血气分析结果，并在入室后、插管前以及插管后使用超声技术测量患者胃窦部横截面积，以评估患者胃进气情况，术中时刻监测血压、心率、脉搏氧饱和度等生命体征。此外，研究人员还要关注低氧血症、恶心呕吐，反流误吸、鼻咽部不适感、气压伤等并发症的情况。

**研究中我该做什么？**

本研究内容嘉兴市第一医院伦理委员会的审查和批准，您需要在我院做一些项目相关检查，在研究期间随时告诉我们您的任何变化或者不适。

**参加该研究将如何影响我的生活？**

您可能会觉得这些检查会带来不便，并且需要特殊的安排。此外，一些检查如行动脉穿刺置管还会使您感觉到不舒服，但这是急诊手术期间常用且重要的监测手段，将保障您在术中的安全；如置入鼻咽通气管可能引发鼻出血，但我们将提前润滑鼻腔和导管，显著降低其发生率，倘若发生一般压迫处理即刻止血。如果您有关于研究中检查和步骤的任何疑问可以向研究医生咨询。

研究期间，您不允许使用未告知研究人员的镇痛药、镇静药、降压药等。您的研究医生会告知您在研究期间：需要配合医生进行诊疗操作，并及时、真实的将自己的情况与反应告知医生，我们将立即给与您反馈和帮助。

**我参加此研究会有什么风险和不良反应？**

研究过程中您可能会出现不良反应。我们会监测研究中所有病人的任何不良反应。如果您出现任何不良反应，请及时向您的研究医生咨询。

目前，项目可能引起的不适：1、低氧血症。2、鼻出血 3、反流误吸等

您需告诉您的家人或与您亲近的朋友您正在参加一项“改良型THRIVE与传统面罩在急诊患者快速顺序麻醉诱导期间的疗效及安全性比较研究”，他们可以注意上面描述的事件。如果他们对您参加研究有疑问，您可以告诉他们怎样联系您的研究医生。

**从此研究中我能得到什么利益？**

参加本研究会使您减少麻醉诱导及插管期间低氧血症的发生，降低二氧化碳蓄积程度，减轻呼吸暂停对您生理状态的干扰。如遇困难插管，还可延长插管安全时间，更好地保障您的生命安全。

**参加本研究会给予我什么报酬？**

您不会因参加本研究而获得任何酬劳，也不会因为参加本研究而获得手术麻醉费用的减免。但凡是本实验所涉及的额外检查及其耗材（腹部超声检查、高流量给氧呼吸机及其呼吸环路、鼻咽通气道、盐酸利多卡因凝胶、石蜡油），我们均将免费提供。

如果我在参加研究期间受到损害会怎样？ 如何避免损害和出现损害后的治疗措施是什么？

理论上，在使鼻咽通气道、HTHRIVE吸氧的情况下患者仍具有低氧血症及相关不良反应的可能。但我们麻醉医师术中管理经验丰富，可根据具体情况个体化处理低氧血症，且手术室供氧设备多样、监护设备完善，发生较长期的低氧血症的可能性是十分小的。但在患者术中合并低血压、水电解质酸碱严重失衡等情况时可能使麻醉管理，因此我们在研究期间将加强循环监测，并且常备血管活性药物等以防治术中并发症。同时我们研究小组将对您的疑问悉心解答，为您提供及时周到的医疗服务。我们也将详细制定方案，严格按照各项规范进行操作。如果您的健康确因参加这项研究而发生与研究相关的损害，请立即通知研究医生，他们将负责对您采取适当的治疗措施。嘉兴市第一医院将承担治疗费用及按国家有关规定对您给予相应的经济补偿。对因医疗事故或因未遵循研究方案程序，或您自身的不良饮食、生活行为而导致的损伤，申办者不予补偿。即使您已经签署这份知情同意书，您仍然保留您所有的合法权利。

**我的个人信息是保密的吗？**

您的医疗记录将保存在医院，研究者、研究主管部门、伦理委员会将被允许查阅您的医疗记录。任何有关本项研究结果的公开报告将不会披露您的个人身份。我们将在法律允许的范围内，保护您个人医疗资料的隐私。

当您签署了这份知情同意书，代表您同意您的个人和医疗信息被用于上述所描述的场合。

我必须参加研究吗？ 有什么替代方案？

参加本研究是完全自愿的，您可以拒绝参加研究，或者研究过程中的任何时候选择退出研究，不需任何理由。该决定不会影响您的正常治疗。

如果您决定退出本研究，请提前通知您的研究医生。为了保障您的安全，您可能被要求进行相关检查，这对保护您的健康是有利的。

我们也为您制定了替代方案，如进行传统加压面罩预给氧、高频喷射通气等进行麻醉诱导及插管期间的供氧。当然，替代方案亦存在相应的风险，比如低氧血症、高碳酸血症、反流误吸等。

受试者同意声明：

我已经阅读了上述有关本研究的介绍，对参加本研究可能产生的风险和受益充分了解。我是自愿同意参加本文所介绍的临床研究。

我同意□不同意□除本研究以外的其他研究利用我的医疗记录和病理检查标本。

知情同意书版本号：1.0 2022年03月10日

受试者签名： 日期：

姓名正楷：

受试者联系电话： 手机号：

法定代表人签名（如有）： 日期：

法定代表人姓名正楷：

研究者声明：我确认已向患者解释了本研究的详细情况，特别是参加本研究可能产生的风险和收益。

研究者签名： 日期：

研究者姓名正楷： 李杰 邓康

研究者联系电话： 13013984418 13736446844

临床科研和医疗新技术伦理委员分会联系方式：浙江省嘉兴市中环南路1882号嘉兴市第一医院，0573-82519988

**Research protocol: part 2**

**Budget**

This study was financially supported by A Project Supported by Scientific Re-search Fund of Zhejiang Provincial Education Department (No. Y202249494), Hangzhou, China and the Construction Project of Anesthesiology Discipline Special Disease Center in Zhejiang (No.201524), Jiaxing, China. The study budget has been allocated as 10,000 RMB for the procurement of NPA, use of HFNO equipment, arterial blood gas analysis and extra drugs for the clinical trial.

**Other support for the project**

Not applicable.

**Collaboration with other scientists or research institutions**

Not applicable.

**Links to other projects**

**Curriculum Vitae of investigators**

Jie Li, M.D., Current Master's degree students at Zhejiang Chinese Medical University, E-mail: **ljljlj4418@163.com**

Bin Liu, M.D., Current Master's degree students at Zhejiang Chinese Medical University, E-mail: [**1114395598@qq.com**](mailto:1114395598@qq.com)

Qing-he Zhou, Ph.D, Professor at Zhejiang Chinese Medical University, Chief physician of The First Hospital of Jiaxing or The Affiliated Hospital of Jiaxing University, Jiaxing, Zhejiang Province 314001, China. E-mail: [**jxxmxy@163.com**](mailto:jxxmxy@163.com)

Hua-dong Ni, Ph.D, Associate professor at Zhejiang Chinese Medical University, Associate Chief Physician of The First Hospital of Jiaxing or The Affiliated Hospital of Jiaxing University, Jiaxing, Zhejiang Province 314001, China. E-mail: [**huadongni@126.com**](mailto:huadongni@126.com)

Ming-juan Liu, M.D., Attending Physician of The First Hospital of Jiaxing or The Affiliated Hospital of Jiaxing University, Jiaxing, Zhejiang Province 314001, China. E-mail: [**liumingjuan163@163.com**](mailto:liumingjuan163@163.com)

Kang Deng, M.D., Associate Professor at Zhejiang Chinese Medical University, Associate Chief Physician of The First Hospital of Jiaxing or The Affiliated Hospital of Jiaxing University, Jiaxing, Zhejiang Province 314001, China. E-mail: [**jxmzdk@163.com**](mailto:jxmzdk@163.com)

**Other research activities of the investigators**

Not applicable.

**Financing and insurance**

Not applicable.
