# Supplementary material for: Pre-oxygenation with high-flow oxygen through the nasopharyngeal airway compared to facemask on carbon dioxide clearance in emergency adults: a prospective randomized non-blinded clinical trial
Source: Eur J Trauma Emerg Surg. 2023 Dec 26;50(3):1051–61. doi: 10.1007/s00068-023-02418-2 (PMC11249433; doi:10.1007/s00068-023-02418-2)
Supplement: Supplementary file 2 — Supplementary file2 (DOCX 806 KB) [file 68_2023_2418_MOESM2_ESM.docx]

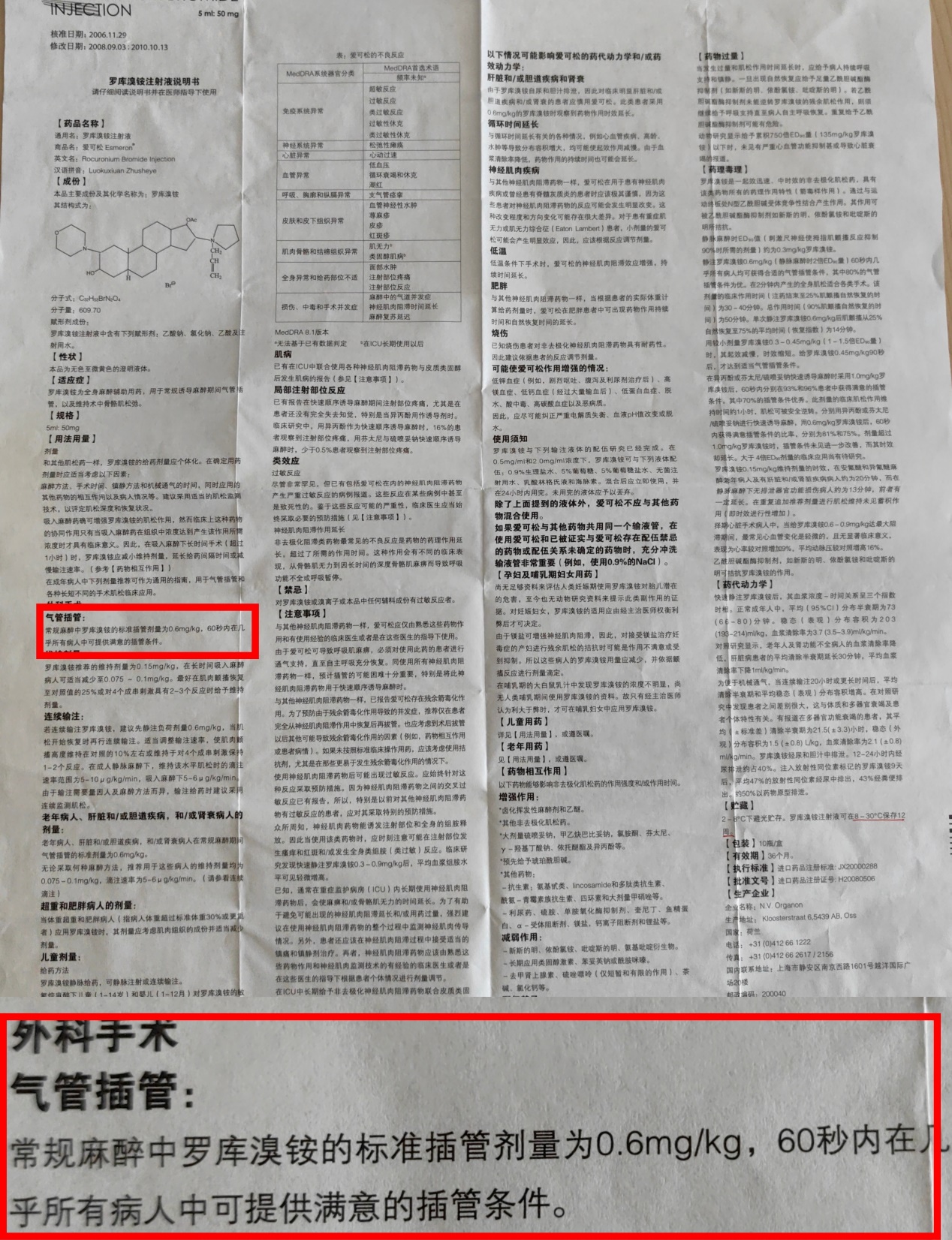


Translation of the highlighted portion: Tracheal Intubation Before Surgery: The standard intubation dose of 0.6 mg/kg of this product in conventional anesthesia provides satisfactory tracheal intubation in most patients within 60 seconds.
