# Supplementary material for: Pre-oxygenation with high-flow oxygen through the nasopharyngeal airway compared to facemask on carbon dioxide clearance in emergency adults: a prospective randomized non-blinded clinical trial
Source: Eur J Trauma Emerg Surg. 2023 Dec 26;50(3):1051–61. doi: 10.1007/s00068-023-02418-2 (PMC11249433; doi:10.1007/s00068-023-02418-2)
Supplement: Supplementary file 3 — Supplementary file3 (DOCX 33 KB) [file 68_2023_2418_MOESM3_ESM.docx]

# Reporting checklist for randomised trial.

Based on the CONSORT guidelines.

|  |  | Reporting Item | Page number |
| --- | --- | --- | --- |
| **Title and Abstract** |  |  |  |
| Title | [#1a](https://www.goodreports.org/reporting-checklists/consort/info/#1a) | Pre-oxygenation with high-flow oxygen through the nasopharyngeal airway compared to facemask on carbon dioxide clearance in emergency adults: a prospective randomized non-blinded clinical trial | 1 |
| Abstract | [#1b](https://www.goodreports.org/reporting-checklists/consort/info/#1b) | **Background:** Before tracheal intubation, it is essential to provide sufficient oxygen reserve for emergency patients with full stomachs. Recent studies have demonstrated that high-flow nasal oxygen (HFNO) effectively pre-oxygenates and pro-longs apneic oxygenation during tracheal intubation. Despite its effective-ness, the use of HFNO remains controversial due to concerns regarding car-bon dioxide clearance. The air leakage and unknown upper-airway obstruction during HFNO therapy cause reduced oxygen flow above the vocal cords, possibly weaken the carbon dioxide clearance.  **Methods:** Patients requiring emergency surgery who had fasted <8 hours and not drunk <2 hours were randomly assigned to the high-flow group, which received 100% oxygen at 30-60 L/min, or the mask group, which received 100% oxygen at 8 L/min. PaO_2_ and PaCO_2_ were measured immediately before pre-oxygenation (T0), anesthesia induction (T1), tracheal intubation (T2), and mechanical ventilation (T3). The gastric antrum’s cross-sectional area (CSA) was measured using ultrasound technology at T0, T1, and T3. Details of complications, including hypoxemia, reflux, nasopharyngeal bleeding, post-operative pulmonary infection, postoperative nausea and vomiting (PONV), and postoperative nasopharyngeal pain, were recorded. The primary out-comes were PaCO_2_ measured at T1, T2, and T3. The secondary outcomes included PaO_2_ at T1, T2, and T3, CSA at T1 and T3, and complications happened during this trial.  **Results:** Pre-oxygenation was administered by high-flow oxygen through NPA (n=58) or facemask (n=57) to 115 patients. The mean (SD) PaCO_2_ were 32.3 (6.7) mmHg in the high-flow group and 34.6 (5.2) mmHg in the mask group (*P*=0.045) at T1, 45.0 (5.5) mmHg and 49.4 (4.6) mmHg (*P*<0.001) at T2, respectively, and 47.9 (5.1) mmHg and 52.9 (4.6) mmHg (*P*<0.001) at T3, respectively. The median ([IQR] [range]) PaO_2_ in the high-flow and mask groups were 404.5 (329.1-458.1 [159.8-552.9]) mmHg and 358.9 (274.0-413.3 [129.0-539.1]) mmHg (*P*=0.007) at T1, 343.0 (251.6-428.7 [73.9-522.1]) mmHg and 258.3 (162.5-347.5 [56.0-481.0]) mmHg (*P*<0.001) at T2, and 333.5 (229.9-411.4 [60.5-492.4]) mmHg and 149.8 (87.0-246.6 [51.2-447.5]) mmHg (*P*<0.001) at T3, respectively. The CSA in the high-flow and mask groups were 371.9 (287.4-557.9 [129.0-991.2]) mm^2^ and 386.8 (292.0-537.3 [88.3-1651.7]) mm^2^ at T1 (*P*=0.920) and 452.6 (343.7-618.4 [161.6-988.1]) mm^2^ and 385.6 (306.3-562.0 [105.5-922.9]) mm^2^ at T3 (*P*=0.173), respectively. The number (proportion) of complications in the high-flow and mask groups is shown below: hypoxemia: 1 (1.7%) vs. 9 (15.8%, *P*=0.019); reflux: 0 (0%) vs. 0 (0%); nasopharyngeal bleeding: 1 (1.7%) vs. 0 (0%, *P*=1.000); pulmonary infection: 4 (6.9%) vs. 3 (5.3%, *P*=1.000); PONV: 4 (6.9%) vs. 4 (7.0%, *P*=1.000), and nasopharyngeal pain: 0 (0%) vs. 0 (0%).  **Conclusion:** Compared to facemasks, pre-oxygenation with high-flow oxygen through nasopharyngeal airway (NPA) offers improved carbon dioxide clearance and enhanced oxygenation prior to tracheal intubation in patients undergoing emergency surgery, while the risk of gastric inflation had not been ruled out. | 1-2 |
| **Introduction** |  |  |  |
| Background and objectives | [#2a](https://www.goodreports.org/reporting-checklists/consort/info/#2a) | Emergency patients fasting for less than the recommended time are at risk of reflux-aspiration. Inappropriate ventilation during induction of anesthesia is assumed to increase this risk Apneic oxygenation during tracheal intubation of general anesthesia in emergency patients with full stomachs is essentially prolonged to safeguard the lives of patients. High-flow nasal oxygen (HFNO), which has been proven to provide effective pre-oxygenation during tracheal intubation, remains controversial in terms of carbon dioxide clearance because of the same size of nasal cannula and anatomical differences in the nasopharynx. | 2-3 |
| Background and objectives | [#2b](https://www.goodreports.org/reporting-checklists/consort/info/#2b) | We delivered high-flow oxygen through nasopharyngeal airway (NPA) to remove obstructive factors and hypothesized that emergency surgical patients accepting high-flow oxygen through NPA for pre-oxygenation would have significant differences in PaCO_2_ and PaO_2_ and not cause reflux or aspiration during tracheal intubation compared to facemasks | 2-3 |
| **Methods** |  |  |  |
| Trial design | [#3a](https://www.goodreports.org/reporting-checklists/consort/info/#3a) | It was a parallel randomized controlled study and participants were assigned in a 1:1 ratio to either the high-flow or mask group. | 3 |
| Trial design | [#3b](https://www.goodreports.org/reporting-checklists/consort/info/#3b) | n/a. We followed a strict schedule of trial and made no changes. | - |
| Participants | [#4a](https://www.goodreports.org/reporting-checklists/consort/info/#4a) | The inclusion criteria were emergency surgical patients of all sexes who were 18-60 years old and had fasted less than 8 hours with a body mass index (BMI) of 18-35 kg m^-2^, and American Society of Anesthesiologists (ASA) class I-III and New York Heart Association (NYHA) class I-II. | 3 |
| Participants | [#4b](https://www.goodreports.org/reporting-checklists/consort/info/#4b) | Data were collected in the Affiliated Hospital of Jiaxing University, Jiaxing, China. | 3 |
| Interventions | [#5](https://www.goodreports.org/reporting-checklists/consort/info/#5) | This trial was actually administered between April 2022 and March 2023.  The high-flow group: An appropriate amount of 2% lidocaine and ephedrine mixed solution (1:200) was dropped into both nostrils by the anesthesiologist. The anesthesiologist selected the NPA, 6.0 mm for females and 6.5 mm for males, and gently inserted it into the nostril on one side after paraffin oil lubrication. The insertion depth was limited to the patient's tolerance, and the target depth (distance from the nose tip to the earlobe) was not required. The NPA was connected to a respiratory line at the interface of a tracheal tube. The high-flow oxygen was started with a flow rate of 30 L min^-1^ and an initial oxygen concentration of 100%. The humidity was set to 100% and the temperature to 37 °C. Patients were asked to breathe deeply for 3 min. After anesthesia induction and patients’ consciousness loss, NPA was placed into the target depth, the oxygen concentration was adjusted to 100%, and oxygen flow was increased to 60 L min^-1^.  Mask group: Anesthesiologists used an anesthetic facemask of an appropriate size to tightly fit the patient's face with a four-head belt. 100% oxygen was delivered at 8 L min^-1^ through a pressure-free circular circuit. The patients were instructed to breathe deeply for 3 min. After patients lost consciousness, the anesthesiologist resolved upper airways collapse and closure by jaw thrust. | 4-6 |
| Outcomes | [#6a](https://www.goodreports.org/reporting-checklists/consort/info/#6a) | The primary outcomes were PaCO_2_ at three separate time points, i.e. immediately before anesthesia induction (T1), tracheal intubation (T2), and mechanical ventilation (T3). The secondary outcomes were PaO_2_ at T1, T2 and T3, and CSA of the gastric antrum at T1 and T3, mean arterial pressure (MAP), and heart rate (HR) at each time-point; occurrence of hypoxemia, reflux aspiration, and nasopharyngeal bleeding during tracheal intubation; and postoperative complications such as pulmonary infection, postoperative nausea or vomiting (PONV), and nasopharyngeal pain. | 7-8 |
| Outcomes | [#6b](https://www.goodreports.org/reporting-checklists/consort/info/#6b) | The changes of PaO_2_ and PaCO_2_ from T0 to T1, T1 to T3 was collected in two groups. The changes of the gastric sinus’ CSA were also discussed. | 7-8 |
| Sample size | [#7a](https://www.goodreports.org/reporting-checklists/consort/info/#7a) | The sample size was determined based on a preliminary experiment (11 participants per group) conducted before the formal start of the study. PaCO_2_ after 3 min of pre-oxygenation was used as the outcome index. According to the pre-experimental results, the mean (SD) PaCO_2_ was 33.9 (6.8) mmHg in the high-flow group and 37.9 (7.2) mmHg in the mask group. A total of 100 patients (50 per group) were required to achieve a power of 90% with a type-1 error of 0.05 for detecting the difference between groups. Considering a 16% dropout rate, at least 116 patients (58 per group) needed to be included in this study. | 6 |
| Sample size | [#7b](https://www.goodreports.org/reporting-checklists/consort/info/#7b) | Participants would drop out because of any result loss, dangerous events or decision to withdraw during study. | 3 |
| Randomization - Sequence generation | [#8a](https://www.goodreports.org/reporting-checklists/consort/info/#8a) | SPSS software version 25 (IBM, Armonk, NY, USA) was used to generate a random sequence that was assigned in a 1:1 ratio to either the high-flow or mask group. | 3 |
| Randomization - Sequence generation | [#8b](https://www.goodreports.org/reporting-checklists/consort/info/#8b) | Simple randomization. A random sequence was assigned in a 1:1 ratio to two groups. | 3 |
| Randomization - Allocation concealment mechanism | [#9](https://www.goodreports.org/reporting-checklists/consort/info/#9) | This sequence was contained in a sealed envelope and randomization was concealed and revealed for the first time after inclusion. | 3 |
| Randomization - Implementation | [#10](https://www.goodreports.org/reporting-checklists/consort/info/#10) | Jie Li generated the allocation sequence, Kang Deng and Jie Li enrolled participants, and Hua-Ding Ni and Ming-Juan Liu assigned participants to interventions | 14 |
| Blinding | [#11a](https://www.goodreports.org/reporting-checklists/consort/info/#11a) | Blinding was not feasible. | 3 |
| Blinding | [#11b](https://www.goodreports.org/reporting-checklists/consort/info/#11b) | n/a. Owing to the nature of the intervention, researchers and participants could not be blind. | - |
| Statistical methods | [#12a](https://www.goodreports.org/reporting-checklists/consort/info/#12a) | Primary and secondary outcomes: Numerical variables were analyzed using an independent samples t-test or the Mann-Whitney U test. Categorical variables were compared using Pearson’s chi-squared test. The primary outcome variables were not all normally distributed, and a non-parametric Mann-Whitney U test was used. Statistical significance was set at *P*<0.05. | 6 |
| Statistical methods | [#12b](https://www.goodreports.org/reporting-checklists/consort/info/#12b) | Outcomes in discussion: Numerical variables were analyzed using t-test or the Mann-Whitney U test. Categorical variables were compared using Chi-square test, Fisher exact test or Pearson’s chi-squared test. Statistical significance was set at *P*<0.05. | 6 |
| **Results** |  |  |  |
| Participant flow diagram (strongly recommended) | [#13a](https://www.goodreports.org/reporting-checklists/consort/info/#13a) | 116 participants were randomly assigned in the high-flow or mask group (58 per group) and received intended treatment, and 115 (58 in the high-flow group and 57 in the mask group) were analyzed finally. | 6 |
| Participant flow | [#13b](https://www.goodreports.org/reporting-checklists/consort/info/#13b) | One patient in the mask group dropped out owing to a suspected allergic reaction. | 7 |
| Recruitment | [#14a](https://www.goodreports.org/reporting-checklists/consort/info/#14a) | From 28 April 2022 to 31 March 2023. | 6, 11 |
| Recruitment | [#14b](https://www.goodreports.org/reporting-checklists/consort/info/#14b) | The trial ended because enough participants had taken part in this trial. | 6 |
| Baseline data | [#15](https://www.goodreports.org/reporting-checklists/consort/info/#15) | A table showing baseline demographic and clinical characteristics for each group had been prepared in the manuscript. | 7 |
| Numbers analysed | [#16](https://www.goodreports.org/reporting-checklists/consort/info/#16) | 58 participants in the high-flow group and 57 in the mask group were included in each analysis and the analysis was by original assigned groups | 6 |
| Outcomes and estimation | [#17a](https://www.goodreports.org/reporting-checklists/consort/info/#17a) | The mean (SD) PaCO_2_ at T1 was 32.3 (3.6) mmHg in the high-flow group vs. 34.6 (5.2) mmHg in the mask group (*P*=0.045); the results for PaCO_2_ at T2 were 45.0 (5.5) mmHg and 49.4 (4.6) mmHg (*P*<0.001); and at T3 were 47.9 (5.1) mmHg and 52.9 (4.6) mmHg (*P*<0.001), respectively.  At T1, the results of the median (interquartile range (IQR) [range]) PaO_2_ were 404.5 (329.1-458.1 [159.8-552.9]) mmHg in the high-flow group and 358.9 (274.0-413.3 [129.0-539.1]) mmHg in the mask group (*P*=0.007). The results for PaO_2_ at T2 were 343.0 (251.6-428.7 [73.9-522.1]) mmHg and 258.3 (162.5-347.5 [56.0-481.0]) mmHg (*P*<0.001), and at T3 were 333.5 (229.9-411.4 [60.5-492.4]) mmHg and 149.8 (87.0-246.6 [51.2-447.5]) mmHg (*P*<0.001) in the high-flow and mask groups, respectively.  The CSA of the gastric antrum were 371.9 (287.4-557.9 [129.0-991.2]) mm^2^ in the high-flow group vs. 386.8 (292.0-537.3 [88.3-1651.7]) mm^2^ in the mask group at T1; *P*=0.920. At T3, the results were 452.6 (343.7-618.4 [161.6-988.1]) mm^2^ vs. 385.6 (306.3-562.0 [105.5-922.9]) mm^2^, respectively (*P*=0.173). In both the high-flow and mask groups, there was no significant difference in CSA between T1 and T3. The CSA measurements were 371.9 (287.4-557.9 [129.0-991.2]) mm^2^ at T1 and 452.6 (343.7-618.4 [161.6-988.1]) mm^2^ at T3 for the high-flow group (*P*=0.081), and 386.8 (292.0-537.3 [88.3-1651.7]) mm^2^ at T1 and 385.6 (306.3-562.0 [105.5-922.9]) mm^2^ at T3 for the mask group (*P*=0.539).  One (1.7%) and nine (15.8%; *P*=0.019) patients had hypoxemia during tracheal intubation in the high-flow and mask groups, respectively. No reflux of gastric contents or nasopharyngeal bleeding was observed in any patient during visual laryngoscopy intubation.  There were no significant differences in the apnea times between the high-flow and mask groups. MAP did not significantly differ between the high-flow and mask groups, nor did HR.  The number (proportion) of patients with pulmonary infection, PONV and nasopharyngeal pain or bleeding in the high-flow and mask groups were 4 (6.9%) vs. 3 (5.3%; *P*=1.000), 4 (6.9%) vs. 4 (7.0%, -2 to 5.9; *P*=1.000), and 0 (0.0%) vs. 0 (0.0%) respectively. | 8-9 |
| Outcomes and estimation | [#17b](https://www.goodreports.org/reporting-checklists/consort/info/#17b) | The number (proportion) of patients who had hypoxemia were one (1.7%) and nine (15.8%; *P*=0.019) patients had hypoxemia during tracheal intubation in the high-flow and mask groups, respectively. No reflux of gastric contents or nasopharyngeal bleeding was observed in any patient during visual laryngoscopy intubation. There were no significant differences in the postoperative complications between the two groups. The number of patients with pulmonary infection, nausea or vomiting and nasopharyngeal pain or bleeding in the high-flow and mask groups were 4 (6.9%) vs. 3 (5.3%; *P*=1.000), 4 (6.9%) vs. 4 (7.0%, -2 to 5.9; *P*=1.000), and 0 (0.0%) vs. 0 (0.0%) respectively | 8-9 |
| Ancillary analyses | [#18](https://www.goodreports.org/reporting-checklists/consort/info/#18) | The magnitude of change in PaCO_2_ during asphyxia (From T1 to T3) was lesser in patients in the high-flow group (15.6 (5.1) mmHg) than in the mask group (18.3 (4.5) mmHg, *P*=0.003).  The mean (SD) increase in PaO_2_ from T0 to T1 was higher in the high-flow group than in the mask group (300.9 (86.1) mmHg in the high-flow group vs. 259.5 (80.2) mmHg in the mask group, *P*=0.009). The median (IQR [range]) decrease in PaO2 from T1 to T3 was lesser in the high-flow group than in the mask group (72.2 (33.7–142.6 [-56.2 to 271.0]) mmHg in the high-flow group vs. 170.3 (116.0-226.3 [-33.8 to 359.2]) mmHg in the mask group, *P*<0.001). | 11-12 |
| Harms | [#19](https://www.goodreports.org/reporting-checklists/consort/info/#19) | The number (proportion) of patients who had hypoxemia were one (1.7%) and nine (15.8%; *P*=0.019) patients had hypoxemia during tracheal intubation in the high-flow and mask groups, respectively. No reflux of gastric contents or nasopharyngeal bleeding was observed in any patient during visual laryngoscopy intubation. There were no significant differences in the postoperative complications between the two groups. The number of patients with pulmonary infection, nausea or vomiting and nasopharyngeal pain or bleeding in the high-flow and mask groups were 4 (6.9%) vs. 3 (5.3%; *P*=1.000), 4 (6.9%) vs. 4 (7.0%, -2 to 5.9; *P*=1.000), and 0 (0.0%) vs. 0 (0.0%) respectively | 10 |
| **Discussion** |  |  |  |
| Limitations | [#20](https://www.goodreports.org/reporting-checklists/consort/info/#20) | Firstly, this is an open trial, as the nature of the intervention did not favor blinding. Secondly, this study could not observe trends in PaO_2_ and PaCO_2_ over an extended period because rapid sequence induction requires minimal respiratory arrest while ensuring adequate sedation and muscle relaxation conditions to reduce the risk of hypoxia. Thirdly, the assessment of significant gas entry into the stomach, facilitated by measuring changes in gastric sinus CSA at different time points, was not continuous and dynamic, permitting possible omissions. | 13 |
| Generalisability | [#21](https://www.goodreports.org/reporting-checklists/consort/info/#21) | Generalisability (external validity, applicability) of the trial findings | 12 |
| Interpretation | [#22](https://www.goodreports.org/reporting-checklists/consort/info/#22) | Patients in the high-flow group basically had higher PaO_2_ than that in the mask group. HFNO generates a certain positive end-expiratory pressure (PEEP) above the glottis through the continuous delivery of high-flow oxygen. The high-flow group could still be provided continuously during tracheal intubation to reduce the rate of PaO_2_ decline.  Our results did indicate additional CO_2_ clearance by high-flow ventilation during apnea. We speculate that the reason for cardiogenic oscillations, acoustic vortex, strong turbulence, flush residual gas from anatomically invalid cavities, Haldane effect and so on.  While our study did not find a statistically significant difference in the CSA at T1 and T3 within each group (*P*=0.081 in the high-flow group and *P*=0.539 in the facemask group), it is important to consider the potential limitations of our research, such as the lack of power. Therefore, there is a potential risk of increased gastric volume during high-flow oxygen through NPA in clinical practice, and further research is needed to investigate the risk of intragastric distension caused by HFNO during preoxygenation. | 3-6 |
| Registration | [#23](https://www.goodreports.org/reporting-checklists/consort/info/#23) | This study was registered at the Chinese Clinical Research Registry (www.chictr.org.cn), ChiCTR2200059192. Name of trial registry is “A study of modified Transnasal Humidification Rapid Insufflation Ventilator Exchange (THRIVE) versus conventional face masks during rapid sequential anesthesia induction in emergency patients”. | 3 |
| **Other information** |  |  |  |
| Interpretation | [#22](https://www.goodreports.org/reporting-checklists/consort/info/#22) | n/a. Refer to appeal interpretations. | - |
| Registration | [#23](https://www.goodreports.org/reporting-checklists/consort/info/#23) | This study was registered at the Chinese Clinical Research Registry (www.chictr.org.cn), ChiCTR2200059192. Name of trial registry is “A study of modified Transnasal Humidification Rapid Insufflation Ventilator Exchange (THRIVE) versus conventional face masks during rapid sequential anesthesia induction in emergency patients”. | 3 |
| Protocol | [#24](https://www.goodreports.org/reporting-checklists/consort/info/#24) | The full trial protocol can be accessed in the Affiliated Hospital of Jiaxing University, Jiaxing, China. | 14 |
| Funding | [#25](https://www.goodreports.org/reporting-checklists/consort/info/#25) | This study was financed by A Project Supported by Scientific Research Fund of Zhejiang Provincial Education Department, China (No.Y202249494) and the Construction Project of Anesthesiology Discipline Special Disease Center in Zhejiang, China (No.201524). | 14 |
|  |  |  |  |
|  |  |  |  |
